# Mutant p53 elicits context-dependent pro-tumorigenic phenotypes

**DOI:** 10.1038/s41388-021-01903-5

**Published:** 2021-11-12

**Authors:** Jennifer J. McCann, Irina A. Vasilevskaya, Christopher McNair, Peter Gallagher, Neermala Poudel Neupane, Renée de Leeuw, Ayesha A. Shafi, Emanuela Dylgjeri, Amy C. Mandigo, Matthew J. Schiewer, Karen E. Knudsen

**Affiliations:** Department of Cancer Biology, Sidney Kimmel Medical College, Philadelphia, PA USA

**Keywords:** Cancer genetics, Prostate cancer

## Abstract

The tumor suppressor gene *TP53* is the most frequently mutated gene in numerous cancer types, including prostate cancer (PCa). Specifically, missense mutations in *TP53* are selectively enriched in PCa, and cluster to particular “hot spots” in the p53 DNA binding domain with mutation at the R273 residue occurring most frequently. While this residue is similarly mutated to R273C-p53 or R273H-p53 in all cancer types examined, in PCa selective enrichment of R273C-p53 is observed. Importantly, examination of clinical datasets indicated that *TP53* heterozygosity can either be maintained or loss of heterozygosity (LOH) occurs. Thus, to mimic tumor-associated mutant p53, R273C-p53 and R273H-p53 isogenic PCa models were developed in the presence or absence of wild-type p53. In the absence of wild-type p53, both R273C-p53 and R273H-p53 exhibited similar loss of DNA binding, transcriptional profiles, and loss of canonical tumor suppressor functions associated with wild-type p53. In the presence of wild-type p53 expression, both R273C-p53 and R273H-p53 supported canonical p53 target gene expression yet elicited distinct cistromic and transcriptional profiles when compared to each other. Moreover, heterozygous modeling of R273C-p53 or R273H-p53 expression resulted in distinct phenotypic outcomes in vitro and in vivo. Thus, mutant p53 acts in a context-dependent manner to elicit pro-tumorigenic transcriptional profiles, providing critical insight into mutant p53-mediated prostate cancer progression.

## Introduction

The tumor suppressor p53 (*TP53*) is the most frequently mutated gene in numerous tumor types, including prostate cancer (PCa), a tumor type that generally exhibits a low mutational burden [1, 2]. PCa remains the second leading cause of cancer-related death in men in the United States [3]. Disseminated, hormone-sensitive PCa (HSPC) is managed with androgen deprivation therapy to target the androgen receptor (AR), but after a median of 24–36 months, the tumor recurs with re-activation of the AR as castration-resistant PCa (CRPC) [4]. Thus, defining the mechanisms that drive progression from HSPC to the currently incurable, metastatic CRPC remains critical. Importantly, *TP53* perturbations were recently shown to outperform *AR* alterations in PCa for predicting negative prognosis [5]. Moreover, genomic analyses of large sequencing cohorts comparing metastatic versus primary disease revealed *TP53* mutations were significantly enriched in metastatic disease, and this was particularly robust in PCa [1, 2]. Mutations in *TP53* have long been implicated as harboring pro-metastatic potential [9], but the functional outcomes of *TP53* mutations in both HSPC and CRPC remain largely unresolved. As such, discerning the mechanisms by which mutant p53 (mut-p53) contributes to tumorigenesis and disease progression in PCa must be addressed.

Recent genomic studies continue to confirm that *TP53* is mutated at high frequencies in cancer [1, 2]. Alterations in *TP53* most commonly occur as missense mutations and map to the DNA binding domain (exons 4–9) at six prevalent hotspots: R175, Y220, G245, R248, R273, or R282 [6–8]. p53 residues R248 and R273 demonstrate the highest frequency of mutation, and expression of tumor-associated mut-p53 at any of these six residues results in a full-length protein with a characteristically extended half-life [6–8]. Mut-p53 is not necessarily functionally equivalent to loss of wild-type p53 (wt-p53), as distinct mut-p53 proteins have been observed to have diverse “gain-of-function” (GOF)-associated pro-tumorigenic roles [8–10]. In particular, R273H-p53 has been shown to associate with the cofactor MRE11 independently of DNA to increase genomic instability, collaborate with transcription factors (e.g. SP1, SREBP, NF-Y) on chromatin to increase proliferation and promote chemo-resistance [11–16], and sequester transcription factors, such as p63, to prevent activation of wt-p53 target genes [15]. Thus, while wt-p53 acts as a tumor suppressor, mut-p53 has the potential to promote tumorigenesis in numerous tumor types through diverse mechanisms, many of which are context-dependent [8, 17]. Various mut-p53 proteins have been shown to drive tumorigenesis, however, the mutational spectrum is diverse and the molecular and biological outcomes of distinct *TP53* mutations have not been thoroughly discerned. Importantly, the context-dependent impact of mut-p53 has yet to be functionally assessed in PCa.

In this study missense mutations in *TP53* were identified as the most frequent mutation across all cancer types examined, including PCa. The majority of *TP53* missense mutations occurred at the R273-p53 residue, a DNA contact point in the p53 DNA binding domain [8]. Across all cancers examined, the R273-p53 residue mutations were similarly distributed between R273C-p53 or R273H-p53, but in PCa R273C-p53 was observed to be selectively enriched with a minor frequency of R273H-p53. Importantly, the samples that harbored mut-p53 retained a wild-type *TP53* allele at a frequency of 55%. Thus, PCa models were generated that express the R273-p53 mutants in the absence of wt-p53 (wt-p53-null) or in the presence of wt-p53, to study the molecular and biological consequences in both clinically relevant contexts. In the wt-p53-null setting, both R273-p53 mutants largely lost the ability to bind chromatin, resulted in similar transcriptional profiles, and demonstrated loss of canonical p53-related target enrichment upon genotoxic insult. Conversely, in the presence of endogenous wt-p53, expression of R273C-p53 and R273H-p53 induced distinct cistromic and transcriptional profiles that supported divergent p53 activity. Moreover, R273C-p53 expression elicited more pro-tumorigenic phenotypes compared to R273H-p53. In sum, these studies reveal context-dependent, pro-tumorigenic functions of p53 missense mutants and for the first time identify R273C as selectively enriched for oncogenic activity in PCa. These findings not only bring new understanding of p53 missense mutants but provide the functional basis for selective enrichment of R273C mutant in PCa.

## Results

### The R273C-p53 allele is selectively enriched in PCa

**Fig. 1:**
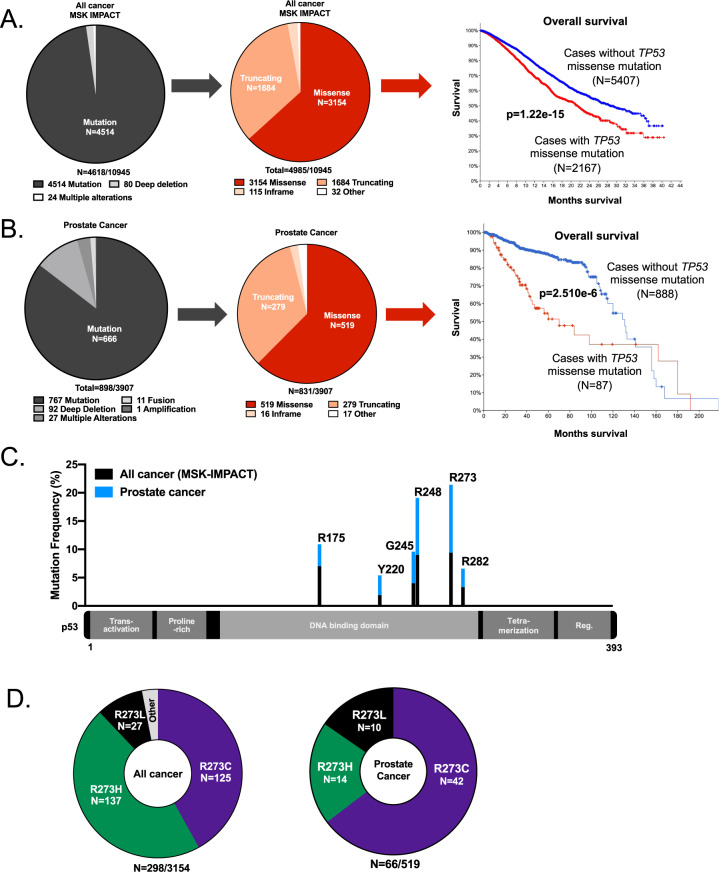
The R273C-p53 allele is selectively enriched in prostate cancer. **A** Across cancer types (MSK-IMPACT), the *TP53* gene is most frequently mutated rather than deleted (left), and these mutations are most commonly missense mutations (multiple mutations can occur in same tumor i.e., *n* = 4618 versus *n* = 4985). Missense mutations in *TP53* are significantly associated with decreased overall survival (*p* = 1.22e−15; MSK-IMPACT study; cBioportal; right). **B** In prostate cancer (cBioportal; see methods for studies used), the *TP53* gene is most frequently mutated compared to having a copy number variation (left), and these mutations are most commonly missense mutations (middle). Missense mutations in *TP53* are significantly associated with decreased overall survival using PCa studies that include survival data (See “Materials and methods” for studies analyzed; *p* = 2.510e−6; right). **C** R273-p53 in the DNA binding domain of *TP53* is the most frequently mutated residue using the studies described in (**A**) for all cancer types (black) and **B** for prostate cancer (blue). The six most frequently mutated residues in *TP53* are shown. **D** In all cancers (MSK-IMPACT), the R273-p53 residue mutations are most frequently observed as an R273C or R273H (left). In PCa studies described in (**B**), R273C-p53 is the most frequently observed (right).

The tumor suppressor gene *TP53* remains the most frequently mutated gene across numerous cancer types, including PCa, even as the number of clinical sequencing datasets continues to increase. However, the diverse functional consequences of distinct *TP53* mutations remain largely unexplained [1, 2]. Through analysis of recently published, prospective clinical sequencing data representing diverse cancer types [1], mutations (missense, truncating, or in frame) were found to be the most frequent *TP53* alterations (*n* = 4514/4618; 97.7%), while copy number changes (*n* = 80/4618; 1.7%), defined as a fusion, amplification, or deletion event (Fig. 1A, left) were found to be less common [1, 8, 18]. The majority of *TP53* mutations were missense mutations (*n* = 3154/4985; 63.3%), suggesting missense mut-p53 could potentially have distinct functional consequences, and hence an evolutionary advantage, compared to *TP53* genomic deletion (Fig. 1A) [1, 8, 18]. Tumors harboring *TP53* missense mutations (*n* = 2167/7574; 28.6%) were significantly associated with decreased overall survival (*p* = 1.22e–15; Fig. 1A, right) compared to tumors without *TP53* missense mutations, also suggesting that *TP53* missense mutations function in tumor progression. As previously reported, *TP53* mutations frequently occur in primary tumors, and are selectively enriched in incurable, metastatic disease [1]. Novel analyses of publicly available PCa studies [19–31] confirmed that mutations in *TP53* were the most frequently observed *TP53* alteration (*n* = 666/898; 74.2%) with a prevalence of missense mutations (*n* = 519/831; 62.5%; Fig. 1B), further suggesting a functional advantage for tumors harboring a missense mutation in *TP53*. Accordingly, missense *TP53* mutations (*n* = 93/982; 9.5%) were significantly associated with decreased overall survival compared to tumors without *TP53* missense mutations (*p* = 2.51e−6; Fig. 1B, right) [20, 24, 25, 28, 32], indicating a potential role for mutant p53 in disease progression.

**Fig. 2:**
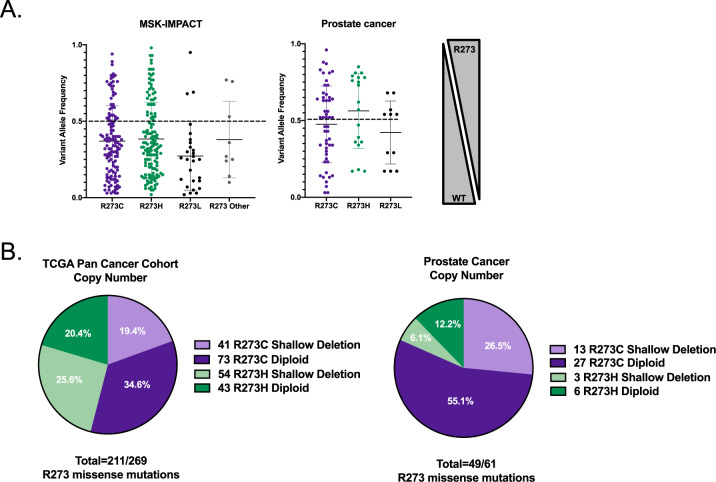
Tumors that harbor a mut-p53 allele often retain wild-type *TP53*. **A** The allele frequencies of *R273C-TP53, R273H-TP53*, and *R273L-TP53* in all cancers (left; MSK-IMPACT) and prostate cancer (right; studies with available allele frequency data—see “Materials and methods”). **B** The copy number changes for *TP53-R273* mutations are observed as both diploid (retaining a wt-p53 allele; heterozygous) or shallow deletion (loss of heterozygosity) using the TCGA Pan Cancer Cohort (left; analyzed January 2020) or all available non-redundant prostate cancer studies—right (see “Materials and methods” for studies used).

Mapping of the most commonly observed missense mutations in *TP53* revealed the R273 DNA contact point residue in the DNA binding domain as the most frequently mutated hotspot (9.45% in all cancers, 22% in PCa; Fig. 1C; Supplementary Fig. 1), further confirming previous studies that demonstrated the R273-p53 residue as frequently altered [8, 18]. Notably, the R273 residue was mutated with similar frequency to an R273C (*n* = 125/298; 41.9%) or R273H (*n* = 137/298; 45.9%) across numerous tumor types, but in PCa, selective enrichment of the R273C-p53 was observed (*n* = 42/66; 63.6%; Fig. 1D, right), suggesting a potential pro-tumorigenic function for R273C-p53 in PCa. Moreover, examination of the allele frequencies in tumor specimens across diverse cancer types revealed that many of the samples harboring R273-p53 mutations occurred in the context of a wt-p53 allele (Fig. 2A, left), as indicated through variant allele frequency (<1). Retention of a WT *TP53* allele was also commonly observed in PCa clinical samples (Fig. 2A, right), demonstrating the importance of studying mut-p53 in the context of wt-p53 expression. Copy number analysis in an additional clinical cohort representing diverse cancer types also demonstrated that R273-p53 mutants frequently retain a WT *TP53* allele (*n* = 116/211; 55.0%; Fig. 2B, left). PCa clinical samples further validated that *TP53* R273 mutant tumors can remain heterozygous (Fig. 2B, right), indicating that ~50% of tumors retain WT *TP53* in the context of a potential gain of function (GOF) R273 allele. These novel findings provide impetus to interrogate the impact of R273-p53 mutants in the absence or presence of wt-p53 expression to more accurately define mut-p53 function in cancer progression.

### R273 mutant p53 proteins lack independent DNA binding activity

**Fig. 3:**
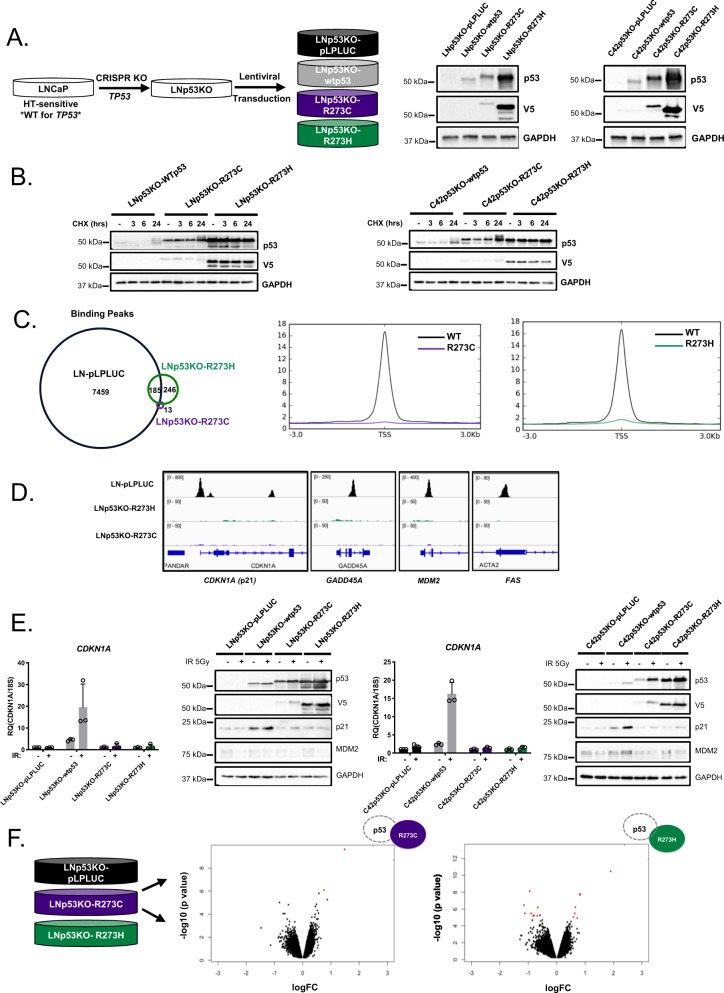
In a p53-null background, R273C and R273H p53 mutants abrogate canonical p53 functions and DNA binding capacity. **A** LNCaP or C4-2 cells underwent CRISPR-mediated *TP53* KO with subsequent, stable expression of control or mut-p53 (left). LNp53KO- and C42p53KO-mut-p53 cells were lysed and immunoblot analysis was performed with the indicated antisera (right) to show mut-p53 expression. **B** LNp53KO- and C42p53KO- mutp53 cells were treated with cycloheximide for the designated time points and immunoblot analysis was performed with the indicated antisera to characterize p53 stability. **C** Chromatin immunoprecipitation sequencing was performed in LN-pLPLUC (wt-p53), LNp53KO-R273C, and LNp53KO-R273H cells in the absence of insult. Shown are the number of p53 binding peaks (left) and binding of p53 ± 3 kb from transcriptional start sites (TSS, right). **D** Binding intensities of p53 peaks observed in chromatin immunoprecipitation sequencing analysis at canonical p53 target genes: *CDKN1A*, *GADD45A*, *MDM2*, *FAS*. **E** LNp53KO- and C42p53KO-mutp53 cells were treated with vehicle or 5 Gy IR, and 4 h post-treatment qPCR analysis of *CDKN1A* expression or immunoblot analysis was performed with the indicated antisera (*N* = 3 independent experiments). **F** Microarray analysis was performed in untreated LNp53KO-pLPLUC, R273C, and R273H cells. Volcano plots demonstrate differentially expressed genes in red (FC > 1.5; *p* < 0.05).

As stated, analyses of recent, publicly available clinical datasets demonstrated that tumors harboring p53 missense mutations either retained (heterozygous) or lost the corresponding WT *TP53* allele (loss of heterozygosity—LOH) at similar frequencies (Fig. 2). To assess the LOH condition, cell models expressing R273-p53 mutants in wt-p53-null background were developed by CRISPR-mediated p53 knockout (p53KO) followed by lentiviral transduction of vector control (pLPLUC), wt-p53, R273C-p53, or R273H-p53 into HSPC or CRPC cells (Fig. 3A; Supplementary Fig. 2A). R273C-p53 and R273H-p53 demonstrated increased protein half-life compared to wt-p53 (Fig. 3B), but largely lost the ability to bind chromatin as determined by p53 chromatin immunoprecipitation sequencing (ChIP-Seq; Fig. 3C). Notably, although both mutants fail to demonstrate independent DNA binding, this loss was more profound for R273C-p53 (13 peaks) than for R273H-p53 (431 peaks, 246 of which were R273H exclusive), suggesting there could be distinct molecular consequences for each mutant. As such, p53 binding at canonical targets was assessed to determine the functional impact of R273C-p53 and R273H-p53 expression. Accordingly, R273C-p53 and R273H-p53 failed to bind to regulatory regions of canonical p53 targets, *CDKN1A, GADD45A, MDM2*, and *FAS* (Fig. 3D). Additionally, induction of the canonical p53 transcriptional target *CDKN1A* (p21) was abrogated in R273C-p53 and R273H-p53 models upon IR at both the mRNA and protein levels (Fig. 3E). Because mut-p53 alone failed to induce canonical p53 targets, unbiased transcriptome analyses were performed to identify any mut-p53 GOF properties. These transcriptomic analyses demonstrated that expression of R273C-p53 or R273H-p53 in the LNCaP-p53KO model induced only modest transcriptional changes (Fig. 3F), with slightly more differential gene expression observed in the R273H mutant (*n* = 21) compared to the R273C mutant (*n* = 2). Accordingly, R273C-p53 and R273H-p53 resulted in similar GSEA pathway enrichment patterns, including enrichment of epithelial to mesenchymal transition (EMT) that has been previously associated with mut-p53 in the wt-p53-null setting (Supplementary Fig. 2B) [33]. Thus, expression of R273C-p53 and R273H-p53 in the absence of wt-p53 does not support canonical p53 target expression and results in comparable transcriptional profiles, suggesting R273C-p53 and R273H-p53 lack independent DNA binding and transcriptional transactivation activity.

### R273C and R273H mutants differentially modify wild-type p53 binding and alter downstream transcriptional networks

**Fig. 4:**
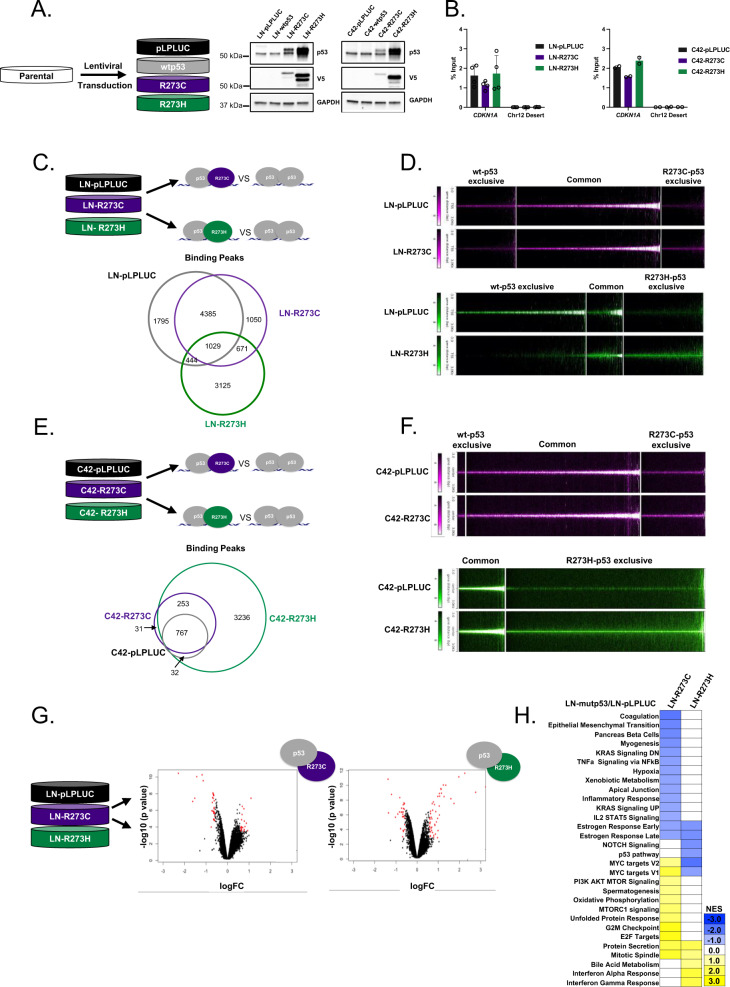
In the presence of wt-p53, R273C and R273H mutants differentially modify p53 binding. **A** LN- and C4-2-derived model systems were made using lentiviral transduction of pLPLUC, wt-p53, p53-R273C-V5, or p53-R273H-V5 constructs into LNCaP or C4-2 cells (left), cells were lysed and immunoblot analysis was performed with the indicated antisera (right). **B** ChIP-qPCR analysis for p53 binding at the *CDKN1A* promoter and chromosome 12 desert (negative control). Biological duplicates or quadruplicates run technical triplicate for each model. **C** Chromatin immunoprecipitation sequencing was performed on LN-pLPLUC, LN-R273C, and LN-R273H cells for p53. The Venn diagram represents the number of p53 binding peaks observed in the designated samples. **D** Binding intensities of p53 in each mut-p53 expressing cell line ±3 kb from transcriptional start sites (TSS) compared to p53 binding in LN-pLPLUC cells (right). **E** Chromatin immunoprecipitation sequencing was performed on C42-pLPLUC, C42-R273C, and C42-R273H cells for p53. The Venn diagram represents the number of p53 binding peaks observed in the designated samples. **F** p53 binding intensities from ChIP-Seq analysis in C42-pLPLUC, C42-R273C, or C42-R273H cells ±3 kb from TSS start sites showing exclusive or common p53 binding by peak intensity designated to each cell line. **G** Microarray analysis was performed in LN-pLPLUC, LN-R273C, and LN-R273H cells in the absence of insult. Significant, differential gene expression is designated in red (FC > 1.5; *p* < 0.05). **H** Hallmark GSEA of LN-R273C and LN-R273H cells compared to LN-pLPLUC for all significantly changed genes observed (*p* < 0.05).

As previously stated, R273-p53 mutants frequently occur in the presence of a WT *TP53* allele in clinical disease (Fig. 2). Thus, to model p53 mutations in the clinically relevant context of wt-p53 expression, R273C-p53 or R273H-p53 was expressed in HSPC and CRPC cells harboring endogenous wt-p53 (Fig. 4A). To ensure that equal copy number of mut-*TP53* was introduced, primers for total *TP53* were designed that recognize both endogenous and exogenous *TP53*. Additionally, endogenous wt-*TP53* primers were designed against the 3ʹ untranslated region (3′ UTR) of endogenous *TP53* (*TP53* 3ʹ UTR), which is absent in the lentiviral constructs (Supplementary Fig. 3A). The data demonstrate that endogenous *TP53* copy number (*TP53* 3ʹ UTR) is identical among all cell lines, and total *TP53* (endogenous + mut-*TP53*; *TP53* total) copy number is elevated in mut-*TP53*-expressing lines due to lentiviral introduction of R273C and R273H mutants (Supplementary Fig. 3B, C, left). Importantly, copy numbers of mut-*TP53* are similar between cell lines in both models, reducing the likelihood of significantly disparate copy number being responsible for possible differences between R273C-p53- and R273H-p53-expressing cell lines (Supplementary Fig. 3B, C, right; mut-TP53 red). A similar assessment of *TP53* using RNA also showed equal expression of mRNA for endogenous *TP53* (*TP53* 3ʹ UTR) and increased total *TP53* expression (*TP53* total; revised Supplementary Fig. 3D, E, left). However, mRNA levels of R273H-p53 were higher than that of p53-R273C in both LNCaP and C4-2 models (mut-*TP53* red; Supplementary Fig. 3D, E, right; Supplementary Fig. 4A). R273C- and R273H-p53 retained the ability to bind chromatin as demonstrated by the binding of p53 to the *CDKN1A* promoter in all wt-p53 expressing cell lines studied (Fig. 4B). Accordingly, in these cells, IR resulted in induced expression of the canonical p53 target gene, *CDKN1A*, as well as p21 and MDM2 protein expression, (Supplementary Fig. 4B), indicating mut-p53 acts in a manner dependent upon expression of wt-p53, and can support wt-p53 functions, in addition to potential GOF activities.

To globally assess p53 binding and mut-p53 GOF potential, ChIP-seq was performed in LN-pLPLUC, LN-R273C, and LN-R273H cells (Fig. 4C). As observed, R273H-expressing cells demonstrated the least number of p53 binding peaks (*n* = 5269), compared to R273C-expressing (*n* = 7135) or control cells (*n* = 7653). While many p53 binding sites were shared among all analyzed cell lines (*n* = 1029), exclusive p53 binding sites were also observed for each condition: LN-pLPLUC (*n* = 1795), LN-R273C (*n* = 1050), and LN-R273H (*n* = 3125; Fig. 4C, D; Supplementary Fig. 4C). Exclusive p53 binding in R273C- or R273H- expressing cells indicates the presence of potential GOF properties that could not only be separate from wt-p53 function but distinct from each other. De novo motif analysis was performed to identify direct p53 binding sites using a minimal 50 bp window in each condition, and the analysis confirmed p53 as the top common motif shared among wt-p53, R273C-p53, and R273H-p53, suggesting mut-p53 cells allow p53-binding at canonical elements (Supplementary Fig. 4D). However, R273C-p53 and R273H-p53 exclusive motifs included a number of additional motifs, indicating an expanded role for R273C-p53 and R273H-p53 in PCa (Supplementary Fig. 4D). Specifically, R273H-expressing cells demonstrated p53 binding to NF-Y and SP2 transcription factor motifs among others that were not bound by p53 in control cells (Supplementary Fig. 4D). Additionally, R273C-expressing cells demonstrated p53 binding to NFAT and SOX2 motifs, which were also not bound by p53 in control cells or R273H-expressing cells (Supplementary Fig. 4D). As a result, R273C- and R273H-expressing cells demonstrated a gain in p53 binding events compared to control, which also differed from each other. To identify potential cofactors that could bind with p53 in mut-p53 expressing cells, known motif analysis was performed using a 1 kb window from each p53 binding site. Consistent with the de novo motif analysis, exclusive putative cofactor binding motifs were observed in LN-R273C cells, including p63, p73, and Elk4, while in LN-R273H cells, motifs for Sp1 and multiple ETS-family transcription factors were observed (Supplementary Fig. 4E, upper panel), supporting a GOF role for both R273-p53 mutants in the presence of wt-p53.

To substantiate and expand on results observed in the LNCaP-derived models, ChIP-seq was also carried out in C4-2-derived cell lines (Fig. 4E, F). As shown, the ChIP-seq analysis demonstrated similar outputs compared to LNCaP-derived models with expanded binding peaks observed in both C42-R273C (*n* = 1339) and C42-R273H (*n* = 4288) cell lines relative to C42-pLPLUC (*n* = 803). The largest number of exclusive binding peaks was observed for C42-R273H cell line (*n* = 3236), similar to LN-R273H cells (Fig. 4C, D). Similar to LN-R273H cells, exclusive known motif analysis of the C42-R273H line showed enrichment of potential binding partners belonging to the ETS family of transcription factors. In addition, enrichment for several FOXA transcription factors, which have been shown to be involved in prostate cancer tumorigenesis [34, 35], was observed in C42-R273H (Supplementary Fig. 4E, lower panel). In sum, while there are more binding sites common for wt-p53 and R273C-p53 in this background (Supplementary Fig. 4F), these data underscore the main conclusion that mutant R273C and R273H exert differential effects in both model systems studied herein.

**Fig. 5:**
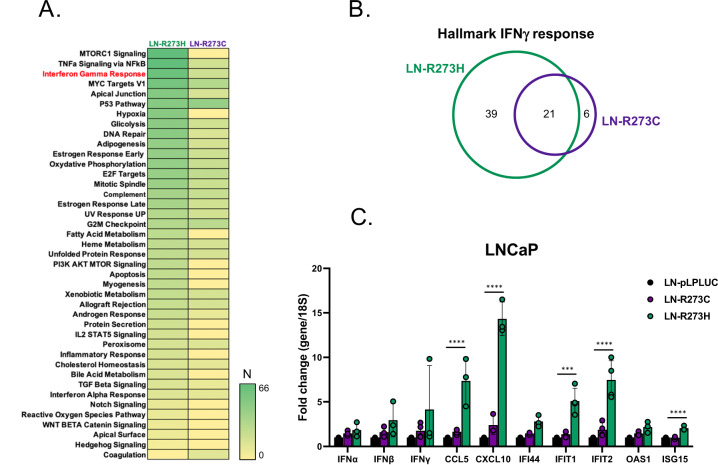
Differential impact of R273C-p53 and R273H-p53 on induction of interferon-regulated genes. **A** Cistrome analysis confirms IFNγ response as one of the top pathways specifically enriched in LN-R273H cell line: GSEA of all genes with TSS within 30 kb of p53 binding peaks specific for LN-R273C and LN-R273H cell lines compared to LN-pLPLUC control. N refers to number of genes enriched in each designated pathway. **B** Venn diagram shows overlap of genes from IFNγ response pathway specific to distinct mut-p53. **C** Expression levels of several representative members of IFNγ response pathway, including the genes identified both in cistrome (*IFIT1* and *ISG15*) and transcriptome (*CXCL10* and *IFIT2*) analyses as specific for LN-R273H cells. Two-way ANOVA test was used to determine statistical significance.

Subsequent transcriptional profiling of LNCaP-derived cells expressing mutant p53 in the presence of endogenous wt-p53 demonstrated differential gene expression for R273C-expressing cells (*n* = 52) or R273H-expressing (*n* = 83) cells compared to control (Fig. 4G). GSEA demonstrated that in this setting, R273C-p53 and R273H-p53 elicited differing transcriptional outputs (Fig. 4H), thus identifying distinct GOF roles for each mut-p53. For example, expression of R273C-p53 resulted in enrichment of several pro-proliferative pathways, including MYC targets, G2M checkpoint, and E2F targets (Fig. 4H), strongly suggesting a pro-tumorigenic role for R273C-p53 in this context. Conversely, expression of R273H-p53 was characterized by de-enrichment of MYC targets (Fig. 4H), indicating a role distinct from R273C-p53. Additionally, these GOF roles appear to be distinct from the p53KO setting (Supplementary Fig. 5). Notably, GSEA of LNCaP mut-p53 expressing transcriptomes revealed specific enrichment of IFNα and IFNγ response pathways in the LN-R273H cell line (Fig. 4H). Moreover, GSEA of genes whose TSS’s were located within 30 kb of p53 binding peaks also indicated IFNγ response as one of the top pathways enriched in LN-R273H cells (Figs. 5A, B). To confirm these data, expression of several genes engaged in IFNγ response was assessed. Results of qPCR analysis showed significant induction of several IFN-regulated genes with known tumor suppressive functions [36–38], specifically in LN-R273H cells (Fig. 5C). Thus, in models of *TP53* heterozygosity, expansion of the p53 cistrome is observed upon expression on mut-p53 in both LNCaP- and C4-2-derived models, and that includes abundant binding sites exclusive to either R273C- or R273H-expressing cells. Accordingly, in the context of wt-p53, R273-p53 mutants elicit distinct transcriptional profiles, with R273C-p53 demonstrating more active involvement in multiple pro-oncogenic pathways compared to R273H-p53, further assuring the probability of distinct p53-regulated downstream events in these PCa models.

### R273C and R273H mut-p53 bind the genome in a manner distinct from the effects of wt-p53 stabilization

**Fig. 6:**
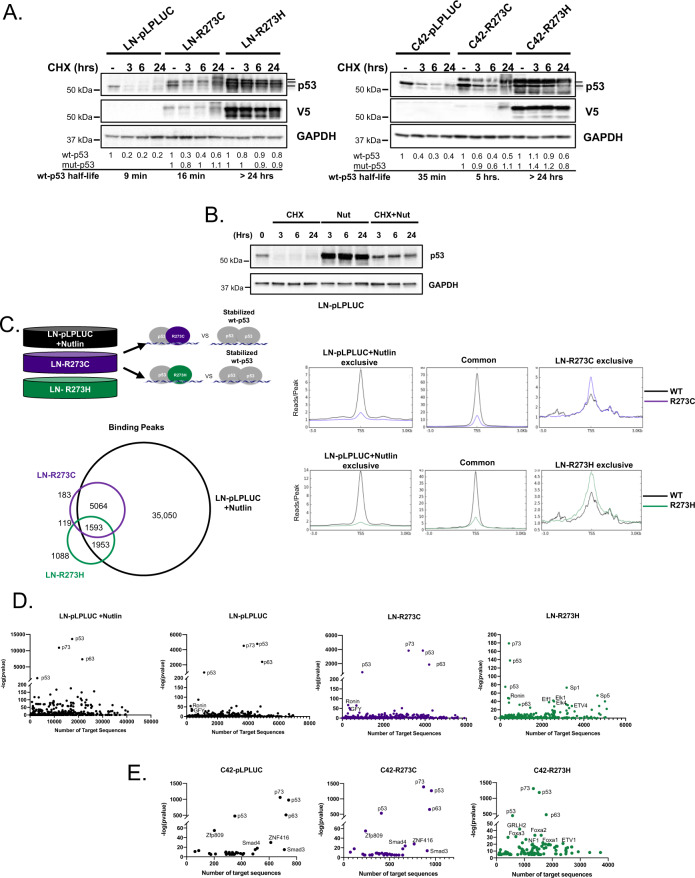
R273C and R273H mutants alter p53 chromatin binding capacity in the manner distinct from the effects of p53-wt stabilization. **A** LNCaP and C4-2 mut-p53 cells were treated with cycloheximide for the designated time and immunoblot analysis was performed with the indicated antisera. Bands corresponding to wt-p53 (lower band), and to mut-p53 (upper bands) are marked. **B** LN-pLPLUC cells were treated as indicated and cells were collected at the designated timepoints to show stabilization of wt-p53 upon treatment with 10 µM nutlin. **C** ChIP-sequencing analysis was performed on LN-pLPLUC + Nutlin (10 μM for 24 h, stabilized wt-p53), LN-R273C, and LN-R273H cells for p53, and the Venn diagram represents the number of p53 binding peaks observed in the designated samples. Binding intensities ±3 kb from the TSS of p53 peaks observed in the designated ChIP-sequencing analysis (right). **D** Known motif analysis of p53 peaks from ChIP-sequencing, using a 1-kb window around the center of binding, for all p53 binding sites in LN-pLPLUC (p53-wt), LN-pLPLUC+Nutlin (stabilized wt-p53), LN-R273C or LN-R273H cells. **E** Known motif analysis of p53 peaks from ChIP-sequencing, using a 1-kb window around the center of binding, for all p53 binding sites in C42-pLPLUC, C42-R273C, or C42-R273H cells.

In the cells modeling heterozygosity, mut-p53 elicited differential binding and transcriptional profiles (Figs. 4, 5). Notably, upon expression of R273C or R273H mut-p53, not only did R273C-p53 and R273H-p53 demonstrate increased protein stability, with a half-life of greater than 24 h, but wt-p53 was also more stable, when compared to control cell lines (Fig. 6A). Thus, to address the question of whether the mut-p53-related expansion of p53 binding was a result of stabilized wt-p53, ChIP-seq was performed using LN-pLPLUC cells expressing endogenous wt-p53 treated with the MDM2 inhibitor, Nutlin-3. As has been well described, Nutlin-3 stabilizes and activates wt-p53 [39–41], and this effect was confirmed here (Fig. 6B). ChIP-Seq analysis comparing p53 binding in LN-R273C and LN-R273H cells to that of LN-pLPLUC cells treated with Nutlin-3 demonstrated the presence of exclusive p53 binding sites in mut-p53 cells (Fig. 6C). As such, despite the tremendous increase in p53 binding in Nutlin-3 treated LN-pLPLUC cells (43,660 peaks versus 7653 in untreated LN-pLPLUC cells), only 183 and 1,088 exclusive p53 DNA binding sites were observed in LN-R273C and LN-R273H cells, respectively. Using de novo motif analysis to directly identify p53 binding in each context demonstrated that while p53 was the top motif in each condition, the majority of binding motifs differed between mut-p53 and stabilized wt-p53 conditions as well as between R273C-p53 and R273H-p53 conditions (Supplementary Fig. 4D). Consistent with the previously discussed motif analysis performed on the wt-p53-null LN-pLPLUC, LN-R273C, and LN-R273H cell lines, R273H-expressing cells maintained exclusive binding to NF-Y and SP2 transcription factor motifs among others that were not bound by Nutlin-activated p53 (Supplementary Fig. 4D). Additionally, R273C-expressing cells demonstrated p53 binding to NFAT and SOX2 motifs, which was absent in the Nutlin-3 treated cells (Supplementary Fig. 4D). Thus, not only does mut-p53 function distinctly from stabilized wt-p53, different mut-p53 proteins perform distinct functions. Accordingly, known motif analysis used to identify putative DNA binding cofactors revealed that R273C-p53 and R273H-p53 differentially support wt-p53 function (Fig. 6D; Supplementary. 4E). For all conditions, the enrichment of p53, p63, and p73 motifs was demonstrated (Fig. 6D, E), but while LN-R273C cells showed motif enrichment of p53, p63, and p73 similar to control, in R273H-expressing cells these motifs were enriched to a much lesser extent compared to either LN-pLPLUC or LN-R273C lines, as indicated by the number of target sequences with p53, p63, and p73-containing motifs (Fig. 6D). Concomitant with a decrease in the number of p53, p63, and p73-containing motifs, many other motifs were enriched in LN-R273H cells, including, Sp1, Sp5, and ETS factors (Fig. 6D). Known motif analysis based on ChIP-seq data from C4-2-derived cells revealed the enrichment of p53, p73, and p63 motifs in all three lines, albeit with lesser number of these motifs in C42-R273H cells, concordant with the results observed in the LNCaP model (Fig. 6D, E). The number of motifs, such as ZNF416, SMAD3, and SMAD4, were also common between control and R273C-expressing lines in both models. Similar to LN-R273H cells, known motif analysis of the C42-R273H line showed enrichment of transcription factors from ETS family (ETV1, ETV4, ELF4, and ETS1 in LN-R273C, Elk1, and Elk4 in LN-R273H). These data show that both p53 mutants, while contributing to stabilization of endogenous wt-p53, exert distinct cancer-promoting features in a manner differing from wt-p53 stabilization, and that varying effects of R273C-p53 and R273H-p53 on dependent transcriptional networks are consistent in both HSPC and CRPC model systems.

### R273C mut-p53 confers more aggressive phenotypes in vitro and in vivo

**Fig. 7:**
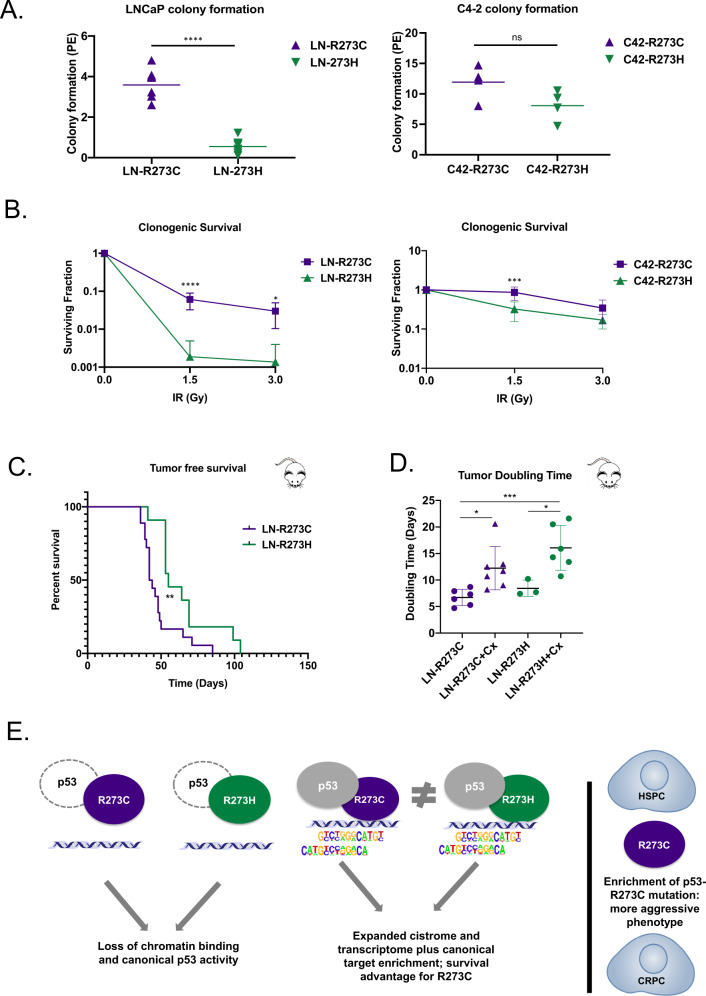
R273C mut-p53 confers more aggressive phenotypes in vitro and in vivo. **A** Colony formation assays were plated using LNCaP- or C4-2-derived cell lines (R273C or R273H) and then harvested to assess colony formation. Graphs represent at least four independent experiments in technical triplicate. Students *t*-test. **B** LNCaP- or C4-2-derived cell lines (R273C or R273H) were plated for clonogenic survival and then treated with the indicated dose of irradiation after 24 h. Graphs represent at least three independent experiments in triplicate. 2-way ANOVA. **C** LN-pLPLUC, LN-R273C, or LN-R273H cells were subcutaneously injected into immunocompromised (Nu/Nu) male mice and the time until tumor take reached 100 mm^3^ or castration surgery was performed between 100–150 mm^3^ (Mantel–Cox Log-Rank; LN-R273C *N* = 18; LN-R273H *N* = 11). **D** The doubling time from tumor take to tumor endpoint was measured in untreated and castrated male mice (one-way ANOVA with Tukey’s multiple comparisons test). **E** Schematic of study findings.

Molecular assessment of R273C-p53 and R273H-p53 suggested distinct functions in the presence of wt-p53 expression, as indicated from the binding profiles as assessed by ChIP-Seq (Fig. 4C, E) as well as preferential enrichment of many pro-tumorigenic pathways in R273C-p53 cells, as demonstrated by GSEA (Fig. 4H). Moreover, R273C-p53 was observed to be specifically enriched in PCa clinical datasets (Fig. 1), indicating a selective advantage of the R273C missense mutation over R273H. To investigate the differential biological consequences of mut-p53 in HSPC and CRPC compared to each other, cell growth and survival assays were performed. While in vitro growth assays did not reveal statistically significant variation in proliferation of cell lines studied (data not shown), evaluation of survival by colony formation assays demonstrated significant differences between R273C- and R273H-expressing cells. Direct comparison demonstrated a 6.5-fold increase in colony formation in LN-R273C cells (*p* < 0.0001) compared to LN-R273H cells (Fig. 7A). C42-R273C cells also formed colonies 1.5-fold more than C42-R273H but did not reach statistical significance (*p* = 0.0982; Fig. 7A). Moreover, LN-R273C cells demonstrated a significant increase in clonogenic survival after 1.5 Gy IR (32-fold; *p* < 0.0001) and 3 Gy IR (22-fold; *p* = 0.019; Fig. 7B). C42-R273C cells demonstrated 2.7-fold increased survival compared to C42-R273H cells after 1.5 Gy IR (*p* = 0.0001; Fig. 7B), indicating a tumorigenic advantage of harboring a R273C mutations in PCa. Accordingly, in vivo subcutaneous xenograft modeling of mut-p53 demonstrated R273C-p53 expression decreased tumor-free survival compared to R273H-p53, defined as the time until the tumor reached 100 mm^3^ or castration surgery was performed (100–150 mm^3^; Fig. 7C). As castration therapy remains the most effective form of therapeutic intervention in HSPC [42], once the tumor reached 100–150 mm^3^, host animals were randomized and subjected to either castration or sham treatment to determine the biological impact of R273C-p53 and R273H-p53 expression in the context of tumor progression. As expected, castration prolonged tumor doubling time compared to the control by 5.5 days for R273C (*p* = 0.04), and 7.6 days for R273H (*p* = 0.02; Fig. 7D). Combined, R273C-p53 demonstrates a selective growth and survival advantage both in vitro and in vivo when compared to R273H-p53.

To summarize, as modeled in PCa, R273C and R273H mut-p53 do not elicit significantly variable molecular outcomes in the context of LOH. Conversely, in the models of heterozygosity supporting endogenous wt-p53 expression, R273C and R273H mut-p53 generate cistromic and transcriptomic outcomes distinct from each other and from the LOH context, resulting in an enhanced pro-oncogenic capacity of R273C-p53 compared to R273H-p53 (Fig. 7E).

## Discussion

Missense mutations, the most common genetic alteration in the tumor suppressor gene *TP53* [1] (Fig. 1), have been linked to metastatic potential and poor prognosis [1, 7, 33, 43]. The functional consequences of distinct mut-p53 alterations, however, remain incompletely understood. Using PCa as a model system, wherein *TP53* is the most frequently mutated gene at a frequency of ~22% (cBioportal), this study used isogenic models to discern how missense p53 mutants differentially affect tumor progression. Data herein advance understanding of the context-dependent mechanisms by which the two most common missense mutants of the hotspot locus R273 (i.e., H and C) contribute to cancer progression with the following conclusions: (i) *TP53* missense mutations are prevalent in PCa with selective enrichment of the R273C-p53 mutation over R273H-p53 occurring in clinical cohorts; (ii) In the absence of wt-p53, R273C-p53 and R273H-p53 are independently insufficient to alter transcriptional networks and show limited DNA binding capacity; (iii) R273C-p53 and R273H-p53 elicit distinct transcriptional enrichment profiles in the presence of wild-type p53 expression, consistent with the observed, expanded mut-p53-defined cistromes; (iv) R273C-p53 promotes aggressive tumorigenic phenotypes compared to R273H-p53 in vitro and in vivo, and is preferentially enriched in advanced PCa. In sum, this study revealed that R273-p53 mutations are distinct and differentially redirect p53 function. These data significantly extend understanding of R273 mutations in p53 and their roles in human disease.

A major advance of the study is that it demonstrates the importance and impact of differential mutant *TP53* selection in cancer. In the clinical setting, there is clear selection for acquisition of R273C-p53 as a function of PCa progression, as compared to other mutations at R273, or as compared to other p53 GOF mutations. This selectivity or enrichment for the R273C mutation is also observed in other genitourinary (GU) cancers, such as bladder urothelial carcinomas (Supplementary Fig. 6A), where, similar to PCa (29 C/13H), the R273C mutation is prevalent (11 C/4H). In addition, mutation to the p53-R273C residue is significantly associated with poor outcome in human disease, as noted by decreased overall survival in patients with an R273C mutation in a bladder and PCa cohort (Supplementary Fig. 6B). Unfortunately, the lack of R273H mutant tumors in this cohort preclude a comparison in this instance, but clinical data from TCGA Pan Cancer Atlas studies (cBioportal) provide evidence of survival disadvantage in patients with R273C p53 mutation compared to R273H (Supplementary Fig. 6C), albeit with some variation between tumor types. For example, shorter overall survival has been demonstrated for patients with R273C-p53 in comparison with R273H-p53 mutation in breast cancer studies (Supplementary Fig. 6D, left), while the opposite was observed in multiple cancer cohorts, including non-small cell lung cancer, glioblastoma, pancreatic adenocarcinoma and ovarian cancer (Supplementary Fig. 6D, right). Given the selection and implications of R273-p53 mutations in cancer progression, it is imperative to understand and model clinically relevant p53-GOF mutations and not assume all mutations, even at the same residue, generate the same molecular and biological outcomes.

Data herein strongly support the contention that the preferentially selected R273C-p53 and R273H-p53 alleles result in distinct outcomes dependent on the presence of a wt-p53 allele. As shown (Fig. 1), R273C/H-p53 is accompanied by a remaining wt-p53 allele in ~55% of tumors. These findings are congruent with recent analyses of numerous clinical cohorts, which call into question the previously held postulate that mutation in one *TP53* allele was typically followed by loss of function of the corresponding WT *TP53* allele [44–46]. At present, genomic analyses available in TCGA (*n* = 10,967) reveal that 51% of tumors with a R273C-p53 or R273H-p53 mutation retain a wild-type allele (*n* = 116/226; 51.3%). This is particularly true in bladder urothelial carcinoma (*n* = 10/16; 62.5%), colon adenocarcinoma (*n* = 55/74; 74.3%), and breast invasive ductal carcinoma (*n* = 31/70; 44.3%), among others (curated set of non-redundant studies; *n* = 47,005; 437 diploid /786 total R273C/H; 55.6%), in addition to PCa. Cistrome and gene expression profiling herein provide mechanistic insight into these observations, as it was observed that neither R273C-p53 nor R273H-p53 showed independent DNA binding capacity or significantly altered downstream gene expression networks in the absence of wt-p53. These data strongly suggest that R273-p53 mutations likely act as loss of function alleles in the absence of wt-p53 and underscore the importance of clinically relevant modeling to discern cancer relevance.

Given these findings, studies presented here utilized novel isogenic models to assess R273C- and R273H-p53 function in the presence of a remaining wt-p53 allele and discovered divergent molecular and biological outcomes. Data obtained herein identified distinct binding events for the two mut-p53 proteins in cooperation with wt-p53, demonstrating 1050 exclusive R273C-related binding sites and 3125 exclusive R273H-related binding sites in the LNCaP model, with similar data for the CRPC model used herein. To our knowledge, this is the first report of direct cistrome mapping of the two clinically relevant mutants in an isogenic system. Data obtained strongly suggest that complexes R273C-p53 are likely to cooperate with a distinct profile of transcription factors to elicit the observed gene expression network. The concept that R273C-p53 preferentially drives p53 complexes toward motifs associated with transcription factors supporting mut-p53 GOF activities, such as ETS or p63/p73, is likely to be shared across multiple tumor types, and is apparent in the ChIP-Seq studies here (Supplementary Fig. 4E). Indeed, previously both R273C-p53 and R273H-p53 have been shown to cooperate with numerous transcription factors, including NF-Y and YAP1, to promote tumorigenesis [15, 16, 47], or sequester tumor suppressive transcription factors p63/p73 to promote disease progression [15, 48], mostly in the LOH context. This study demonstrated that R273C-p53 retained the ability to associate with putative cofactors in concert with wt-p53 expression. Known motif analysis revealed different presumed cofactors for R273C and R273H p53 mutants in both models: R273C-p53 was shown to preferentially associate with p63/p73, while R273H-p53 preferentially associated with multiple transcription factors of ETS family (Supplementary Fig. 4E), which has been observed for R273H-p53 in breast cancer models [49]. More importantly, de novo motif analysis of LNCaP-derived cell lines demonstrated that R273C-p53 had the ability to drive wt-p53 to RXR, NFAT, and Sox2 motifs (Supplementary Fig. 4D), which were not observed in control or R273H-p53 expressing lines, with clinical implications for PCa. As such, modulation of the RXR-related hormone receptor pathway has been shown to affect PCa cell proliferation [50]. Nuclear factor of activated T cells (NFAT) mediates PCa proliferation through activation of Ca^2+^-dependent NFAT transcription [51, 52]. Additionally, Sox2 was identified to be a critical mediator of lineage specificity in PCa cells in the context of p53 null and RB null tumors [53], and was shown to promote CRPC [54]. These newly identified R273C-p53 co-factors are of particular interest in future studies. Thus, R273C-p53 differentially associates with cofactors, like p63/p73, to elicit pro-tumorigenic phenotypes, while driving wt-p53 to novel DNA binding sites.

Strikingly, these data not only identify novel functions for R273C-p53, but underscore differences from other reported GOF mutations. First, distinctions are noted with regard to the impact of GOF alleles in the presence and absence of wt-p53. Whereas in a previous study, R175H, R248Q, and R273H mut-p53 demonstrated significantly attenuated DNA binding capacity in the wt-p53 null context [55], R248Q-p53 proved functionally equivalent in the maintenance of heterozygosity and LOH setting and acted in a strong, dominant negative manner to the wt-p53 allele [55]. Another study focused on breast cancer models found that while R273H-p53 regulated PARP, YAP, and paxillin localization to the cytoplasm, the mut-p53 proteins R280K-p53 and L194F-p53 did not, indicating that different hotspot mutants elicit distinct functions [56]. These combined findings highlight the importance of understanding clinical prevalence and modeling of individual p53 GOF alleles according to observation. Second, distinctions are observed with regard to binding and putative cooperative factors. As was shown previously in various (non PCa) tumor models, R175H-p53, R248W-p53, and R273H-p53 have been able to partner with YAP, NF-Y, and p73 factors; R248W-p53 mutant also demonstrated strong association with SP1 and ETS2, and R273H-p53 cooperated with CBP, SREBP and NRF2 [45]. While some of the previously established co-factors were identified in this study via ChIP-Seq (i.e., NF-Y, Sp1, p63/p73), others, such as SREBP2, TOPBP1, and MRE11, were not identified, emphasizing further importance of context specificity [8]. Finally, distinctions are noted with regard to resulting downstream gene networks. While downstream transcriptional networks have been characterized for R273H-p53 in several tumor models, transcriptional networks related to R273C-p53 remain largely unknown [8]. As such, this study provides novel insight in the R273C-p53-dependent transcriptome, which differs from R273H-p53, most significantly in enrichment of E2F targets, further indicating a potential role for R273C mutant in lineage specificity, perhaps in the context of RB loss. Additionally, metabolic processes, such as oxidative phosphorylation, proliferative, and cell cycle pathways were also enriched upon R273C-p53 expression. The up-regulation of genes engaged in IFNγ response, specifically in LN-R273H cells, is also significant. As augmented expression of these genes was observed in the cell line demonstrating less aggressive phenotype in vitro and in vivo, this may be relevant to the reduced pro-oncogenic potential of R273H-expressing cells. These data further underscore the differential impact of R273C versus R273H in downstream networks of tumor relevance, which could confer a survival advantage of R273C harboring tumors over R273H. R273C induces pro-tumorigenic gene networks in common with R273H but in the absence of interferon response induction, which can involve multiple intermediates with known tumor suppressive functions [36–38]. Future studies are needed to discern transcriptional impact of these observations and to further understand how distinct p53 mutations drive tumor progression.

Finally, the data herein link R273C-p53 to aggressive phenotypes. As shown, R273C-p53 was associated with enhanced tumor growth and survival as compared to R273H-p53, using both in vitro and in vivo models. These findings are concordant with the observed clinical selection for R273C-p53 as a function of disease progression and provide the basis for future investigation of R273C-p53 function. Based on the current findings, increased tumorigenicity associated with the R273C-p53 allele may be linked to a number of putative downstream activities. For example, as described above, de novo motif analysis (Supplementary Fig. 4) revealed enrichment of potential cooperative factors that exert known roles in cancer progression (e.g., HIC2, KLF3, SOX2) [54]; the relative impact of these likely contributing factors should be assessed. Relatedly, R273C-p53 demonstrates significantly higher putative association with the p73/p63 transcription factors than R273H-p53. The formation of the mut-p53/p63 or mut-p53/p73 complexes inhibits the related tumor suppressive functions [15, 16, 47] suggesting that modification of activity may promote aggressive features of R273C p53 mutant. Finally, it is formally possible the mutation may invoke differences in cytoplasmic functions of p53 mutants. Since cytoplasmic wt-p53 regulates multiple cellular processes including cell death pathways, such as apoptosis and autophagy [57], it is plausible to assume that p53 mutants accumulated in the cytoplasm could interfere with these p53 activities by either forming aggregates with wt-p53 or competing for the binding to its cytoplasmic partners. However, the studies providing rigorous experimental evidence are scarce. As such, there are data implicating cytoplasmic R273H-p53 in inhibition of autophagy [58], which depending on context can be both onco-suppressive or tumor-promoting [59]. It has been also shown that although R273H-p53 accumulates and localizes to mitochondria, it does not interact with anti-apoptotic Bcl2-family members and, consequently, is unable to induce intrinsic apoptosis [60, 61]. Additionally, R273H-p53 previously was shown to alter cellular localization of PARP1, a critical mediator of PCa progression [56] and to affect pro-inflammatory TNF signaling [62]. Notably, there are no studies ascertaining cytoplasmic functions of R273C-p53, and its impact remains to be investigated. While the relative contribution of these putative R273C-p53 functions to the observed enhanced survival of R273C-p53 expressing cells in vitro and in vivo has yet to be fully discerned, these findings clearly demonstrate that mutant p53 proteins differentially impact tumor outcomes.

In summary, the studies herein reveal the differential molecular, cellular, and in vivo impact of distinct R273-p53 mutants in cancer (Fig. 7E) and lay the foundation for discerning the role of the relevant R273C-p53 mutant in conferring poor outcome.

## Materials and methods

### Cell lines, cell culture, and maintenance

All LNCaP and C4-2 derived cell lines were maintained in minimum essential media (IMEM) supplemented with 5% FBS (Heat inactivated fetal bovine serum) with 2 mmol/L of L-glutamine and 100 units/ml penicillin–streptomycin. LNCaP and C4-2 cell lines were authenticated by ATCC and checked for mycoplasma upon thawing and termination of maintenance (<20 passages) using the ATCC Universal Mycoplasma Detection Kit. For generation of stable cell lines, LNCaP and C4-2 cells were transduced with lentivirus and underwent at least three rounds of selection with the appropriate antibiotic. LNCaP and C4-2 cells underwent *TP53* knockout using the Santa Cruz p53 CRISPR/Cas9 KO plasmid and p53 HDR plasmid following the manufacturer’s instructions (Santa Cruz Biotechnology). Briefly, cells were seeded to be ~50% confluent on the day of transfection in antibiotic-free media. CRISPR/Cas9 *TP53* KO plasmid (sc-416469) and the associated HDR plasmid (sc-416469-HDR; Santa Cruz Biotechnology) were co-transfected, and 48 h post-transfection cells began selection with puromycin.

### Plasmids

All plasmids were developed using Gateway cloning technology (ThermoFisher). The following expression plasmids were used: pLenti4/TO/V5-pLPLUC (vector control expressing luciferase), pLenti4/TO/V5-R273C, pLenti6/V5-p53_R273H (Addgene plasmid #22934) and pLenti6/V5-p53_wt p53 (Addgene plasmid #22945) were gifts from Bernard Futscher [63].

### Western blotting

Cell lysates were prepared in RIPA buffer and three rounds of sonication were performed for each sample (30 s on, 30 s off) in Bioruptor Pico (Diagenode). The following antibodies were used: p53 (DO-1) sc-126 Santa Cruz; MDM2 sc-965 Santa Cruz; p21 Abcam ab109520; GAPDH sc-25778 Santa Cruz; V5 Invitrogen R960-25; Cycloheximide was used as described (Sigma Aldrich). Band densities for wt-p53 and mut-p53 (Fig. 5A) were measured using ImageJ software, normalized to GAPDH content, and presented as fold increase compared to untreated control.

### Gene expression

Cells were treated as indicated, and RNA was isolated using TRIzol (Life Technologies). cDNA was synthesized using Superscript VILO cDNA Synthesis Kit (Thermo Fisher), following the manufacturer’s instructions. RT-PCR was performed in biological triplicate and technical quadruplicates on a Step One Plus Real Time PCR machine (Applied Biosystems) using SYBR Green master mix (Thermo).

### Copy number variation analysis

Untreated cells were collected for DNA and RNA isolation in biological triplicate. DNA was isolated using DNeasy Blood and Tissue Kit (#69504) with incorporated RNAse A treatment, according to manufacturer recommendations. 30 ng of DNA was used per qPCR reaction. RNA was isolated with RNeasy Mini Kit (Qiagen, #74104) incorporating in-column DNAse treatment using RNase-Free DNase Set (Qiagen, #79254) following manufacturer procedures. cDNA was synthesized as described above. qPCR was performed on DNA and cDNA templates using primers against total TP53 (TP53) and endogenous wt-TP53 (TP53 3’UTR) described below.

### Primers

The following primers were used for quantitative PCR:

**Table:** 

Target	Forward Sequence (5’ to 3’)	Reverse Sequence (5′ to 3′)
18S	GCAATTATTCCCCATGAACG	GGCCTCACTAAACCATCCAA
TP53	CCCAAGCAATGGATGATTTGA	GGCATTCTGGGAGCTTCATCT
TP53 3′UTR	CAGTCTACCTCCCGCCATAA	GCTGTCAGTGGGGAACAAGA
CDKN1A	GGCAGACCAGCATGACAGATT	GCGGATTAGGGCTTCCTCT
IFNα	ACTCATACACCAGGTCACGC	TGGTCATAGTTATAGCAGGGGTG
IFNβ	GCTTGGATTCCTACAAAGAAGCA	ATAGATGGTCAATGCGGCGTC
IFNγ	TCGGTAACTGACTTGAATGTCCA	TCGCTTCCCTGTTTTAGCTGC
CCL5	CCAGCAGTCGTCTTTGTCAC	CTCTGGGTTGGCACACACTT
CXCL10	AGTGGCATTCAAGGAGTACC	TGATGGCCTTCGATTCTGGA
IFI44	GATGTGAGCCTGTGAGGTCC	CTTTACAGGGTCCAGCTCCC
IFIT1	TTGATGACGATGAAATGCCTGA	CAGGTCACCAGACTCCTCAC
IFIT2	AAGCACCTCAAAGGGCAAAAC	TCGGCCCATGTGATAGTAGAC
OAS1	TGTCCAAGGTGGTAAAGGGTG	CCGGCGATTTAACTGATCCTG
ISG15	CTCTGAGCATCCTGGTGAGGAA	AAGGTCAGCCAGAACAGGTCGT
*ChIP-qPCR*		
Chr12 Desert	GGGATGATGTGTGGGTTTTACC	CAATATCCAGCGAAAAGGAAGCT
CDKN1A	AGCAGGCTGTGGCTCTGATT	CAAAATAGCCACCAGCCTCTTCT

### Microarray analysis

LN- and LNp53KO- pLPLUC, wt-p53, R273C, and R273H cells plated for 48–72 h in biological triplicate. Cells were treated as indicated, and RNA was isolated using TRIzol (Life Technologies) with an additional ethanol precipitation using 3 M sodium acetate to remove phenol contamination. Proper RNA integrity was confirmed using an Agilent Bioanalyzer in the SKCC Cancer Genomics and Bioinformatics Core. Gene expression was profiled using the Affymetrix Human Transcript 2.0 microarray, with hybridization performed using the GeneChip Hybridization Oven 645, followed by scanning on Affymetrix Gene Chip Scanner 3000. Data preprocessing was performed in R using RMA normalization. Microarray probe sets were filtered and only probe sets annotated with an official gene symbol were included for further analysis. Microarray data have been deposited in the GEO repository under the accession number GPL17586. All GEO-associated accessions from this study, including ChIP-Seq and microarray, can be located using GSE157337.

### Chromatin Immunoprecipitation (ChIP)-sequencing

LN-pLPLUC, LN-R273C, LN-R273H, and LN-pLPLUC+Nutlin samples were prepared in biological duplicate and the input samples were pooled. Sample preparation and analysis was performed as previously described [64, 65]. In brief, cells were crosslinked with fresh 1% formaldehyde for 10 min at room temperature. Chromatin was sheared to ~200 base pairs using a Diagenode Ultrasonicator for at least 30 cycles (30 s on, 30 s off). Antibody used for ChIP was p53 FL-393 (Santa Cruz, sc-6343). The libraries were constructed using the Swift BioSciences ACCEL-NGS 2 S Plus DNA Library kit with approximately 10 ng of DNA. The Illumina NextSeq 500 was used to sequence samples at the TJU Sidney Kimmel Cancer Sequencing Core Facility. ChIP-seq was aligned using Bowtie2 (v2.3.2) [66]. Peak calling was performed using MACS2 (v2.1.1) with a q-value cutoff of 0.05 [67]. Profiles and heat-maps were generated using DeepTools (v2.5.7) [68]. Motif enrichment performed using Homer (v4.10.3) [69]. ChIP-seq of C42-pLPLUC, C42-R273C and C42-R273H cells was performed in identical manner, except the antibody used for C4-2 ChIP was from Bioss (p53 FL-393, bc-8687R), as sc-6343 is no longer commercially available. Both antibodies are directed against the same epitope. ChIP-Seq data have been deposited in the GEO repository under the accession number GSE157335. All GEO-associated accessions from this study, including ChIP-Seq and microarray, can be located using GSE157337.

### Clonogenic survival assays

Clonogenic assays were performed as described previously [70, 71]. Briefly, cells were counted and plated in triplicate in 12 well plates at different densities (2 × 10^3^ and 4 × 10^3^ for HSPC lines, and 1 × 10^3^ and 2 × 10^3^ for CRPC lines). Cells were either left untreated or were subjected to 1.5 Gy or 3 Gy of ionizing irradiation (IR) 24 h after plating. 14 (C4-2) or 21 (LNCaP) days post-treatment cells were fixed in 0.5% crystal violet with 37% formaldehyde in PBS. Aggregates of more than 50 cells, as determined by microscopy, were counted as clones. Plating efficiency (PE) was calculated from untreated cells ((No. colonies/No. cells seeded) × 100). Survival was calculated by ((No. colonies formed after treatment)/(No. cells seeded) × PE). All experiments were performed at least three times.

### In vivo studies

Mice were housed in animal facilities within the Sidney Kimmel Cancer Center at Thomas Jefferson University, and all protocols used for this study were approved by the Institutional Animal Care and Use Committee (IACUC) at Thomas Jefferson University. Xenograft studies were performed in accordance with NIH Guidelines, and animal protocols were approved by Institutional Animal Care and Use Committee at Thomas Jefferson University. Cells (3 × 10^6^ per injection) suspended in PBS were combined 1:1 with Matrigel (BD Biosciences; 354234) and injected subcutaneously into the left flank of male NCI Ath/Nu mice aged 34–42 days (Charles River Laboratories). At least ten mice were injected with each cell type to ensure at least three mice could be randomized into treatment groups. The investigator was not blinded to treatment group allocation. Tumor development was monitored over time by palpation and caliper measurements. Once a tumor reached 100–150 mm^3^, mice were randomized and subjected to either castration or sham treatment as previously described [72]. No specific method of randomization was used, but mice were placed in treatment groups as tumors developed, typically in a 1:1 ratio. Tumor volume was measured 3 times per week, and mice were sacrificed when the tumor reached ~1000 mm^3^. Animals were excluded from tumor doubling time analysis if their tumor was greater than 150 mm^3^ at the time of tumor take, had to be sacrificed before the experimental endpoint was reached, or if a tumor did not develop.

### Statistical analysis

In vitro and In vivo data are presented as mean ± standard deviation, unless otherwise indicated. Statistical analyses were performed using GraphPad Prism 8.

### Analysis of clinical datasets (cBioportal [73, 74])

All data used from the designated cBioportal studies was downloaded March 2019, unless otherwise indicated. For all cancer types, the MSK-IMPACT study was used [1]. The PCa studies used for Fig. 1 included: MCTP, Nature 2012 [19], SU2C/PCF Dream Team, PNAS 2019 [20], SU2C/PCF Dream Team, Cell 2015 [21], Multi-Institute, Nat Med 2016 [22], Broad/Cornell, Cell 2013 [23], Broad/Cornell, Nat Genet 2012 [24], CPC-GENE, Nature 2017 [25], EurUrol, 2017 [26], Fred Hutchinson CRC, Nat Med 2016 [27], MSKCC, Cancer Cell 2010^28^, MSKCC/DFCI, Nature Genetics 2018 [29], TCGA, PanCancer Atlas, MSKCC, Cell 2014 [30], MSKCC, JCO Precis Oncol 2017 [31], The Metastatic PCa Project (Provisional, December 2018). Figure 1B, right (analyzed August 2020): CPC-GENE, Nature 2017 [25], Broad/Cornell, Nat Genet 2012 [24], MSKCC, Cancer Cell 2010^28^, MSKCC, PNAS 2014 [32], SU2C/PCF Dream Team, PNAS 2019 [20], TCGA, PanCancer Atlas. Figure 2A, right (analyzed August 2020): MSK, Clin Cancer Res 2020 [75], SU2C/PCF Dream Team, PNAS 2019 [20], SU2C/PCF Dream Team, Cell 2015 [21], MSK, Eur Urol 2020 [76], MSKCC/DFCI, Nature Genetics 2018 [29], Fred Hutchinson CRC, Nat Med 2016 [27], MCTP, Nature 2012 [19], Multi-Institute, Nat Med 2016 [22], TCGA, PanCancer Atlas. Figure 2B, right (analyzed August 2020): Multi-Institute, Nat Med 2016 [22], MSK, Eur Urol 2020 [76], Fred Hutchinson CRC, Nat Med 2016 [27], MSK, Clin Cancer Res 2020 [75], MCTP, Nature 2012 [19], MSKCC, JCO Precis Oncol 2017 [31], SU2C/PCF Dream Team, PNAS 2019 [20], MSKCC/DFCI, Nature Genetics 2018 [29], SU2C/PCF Dream Team, Cell 2015 [21], Broad/Cornell, Cell 2013 [23], MSKCC, Cancer Cell 2010 [28], TCGA PanCancer Atlas. Duplicate mutations in patient samples were not counted.

## Supplementary information

Supplemental Figures
