# Supplementary material for: Mutant p53 elicits context-dependent pro-tumorigenic phenotypes
Source: Oncogene. 2021 Nov 12;41(3):444–58. doi: 10.1038/s41388-021-01903-5 (PMC8755525; doi:10.1038/s41388-021-01903-5)
Supplement: Supplementary file 1 — Supplemental Figures [file 41388_2021_1903_MOESM1_ESM.pptx]

## Slide 1
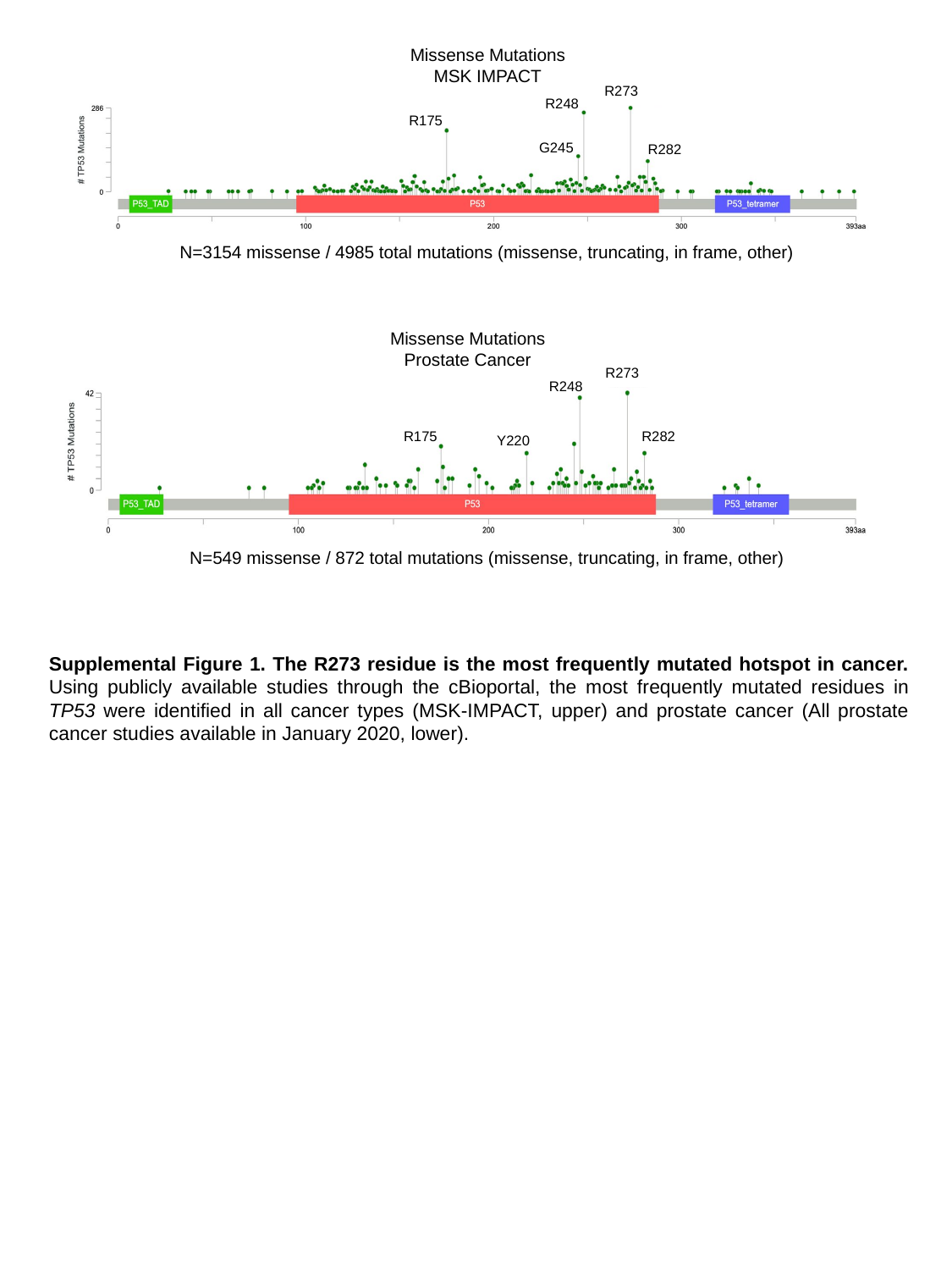

Missense Mutations
MSK IMPACT
R273
R248
R175
G245
R282
N=3154 missense / 4985 total mutations (missense, truncating, in frame, other)
Missense Mutations
Prostate Cancer
R273
R248
R175
R282
Y220
N=549 missense / 872 total mutations (missense, truncating, in frame, other)
Supplemental Figure 1. The R273 residue is the most frequently mutated hotspot in cancer. Using publicly available studies through the cBioportal, the most frequently mutated residues in TP53 were identified in all cancer types (MSK-IMPACT, upper) and prostate cancer (All prostate cancer studies available in January 2020, lower).

## Slide 2
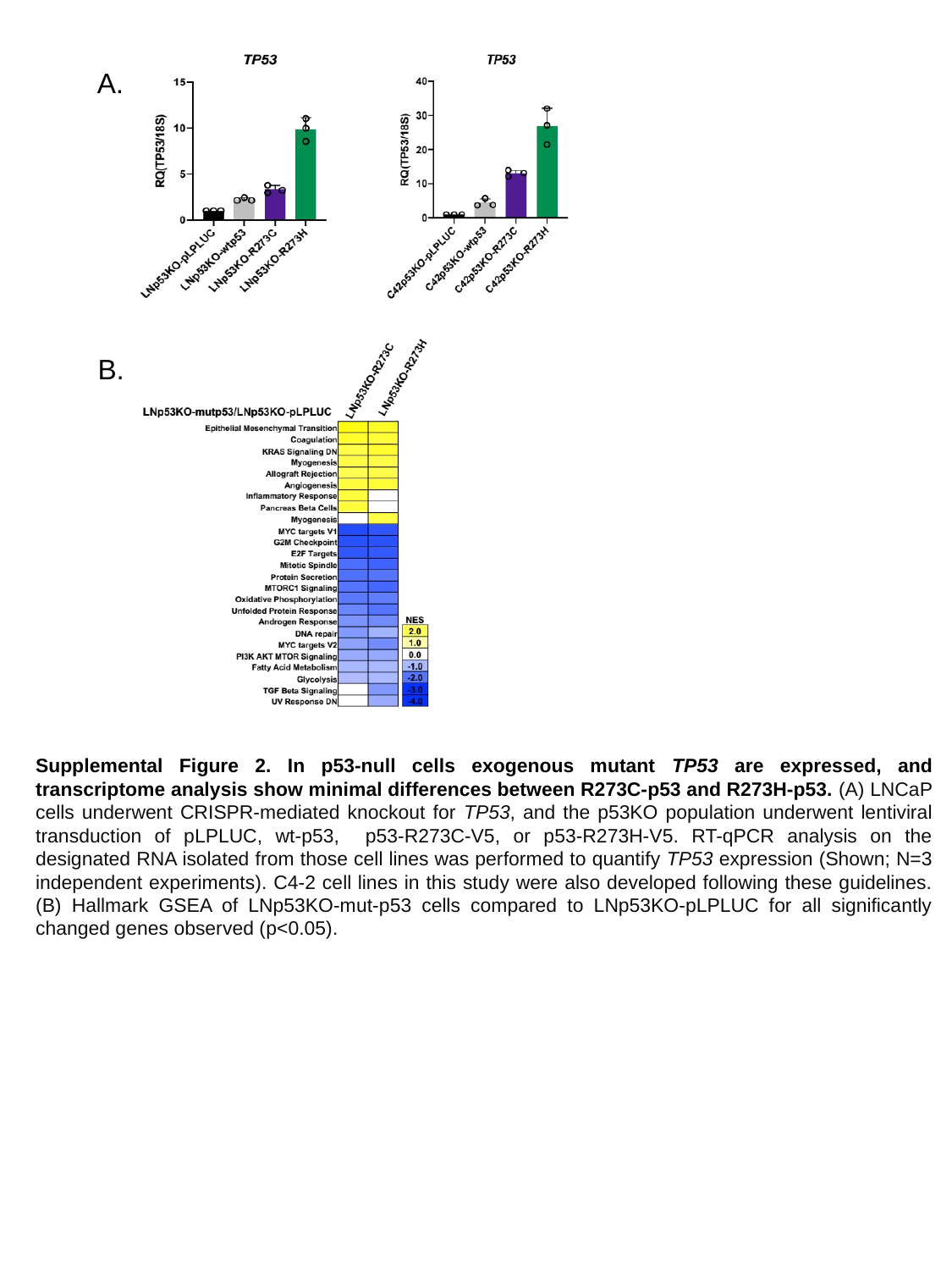

A.
B.
Supplemental Figure 2. In p53-null cells exogenous mutant TP53 are expressed, and transcriptome analysis show minimal differences between R273C-p53 and R273H-p53. (A) LNCaP cells underwent CRISPR-mediated knockout for TP53, and the p53KO population underwent lentiviral transduction of pLPLUC, wt-p53, p53-R273C-V5, or p53-R273H-V5. RT-qPCR analysis on the designated RNA isolated from those cell lines was performed to quantify TP53 expression (Shown; N=3 independent experiments). C4-2 cell lines in this study were also developed following these guidelines. (B) Hallmark GSEA of LNp53KO-mut-p53 cells compared to LNp53KO-pLPLUC for all significantly changed genes observed (p<0.05).

## Slide 3
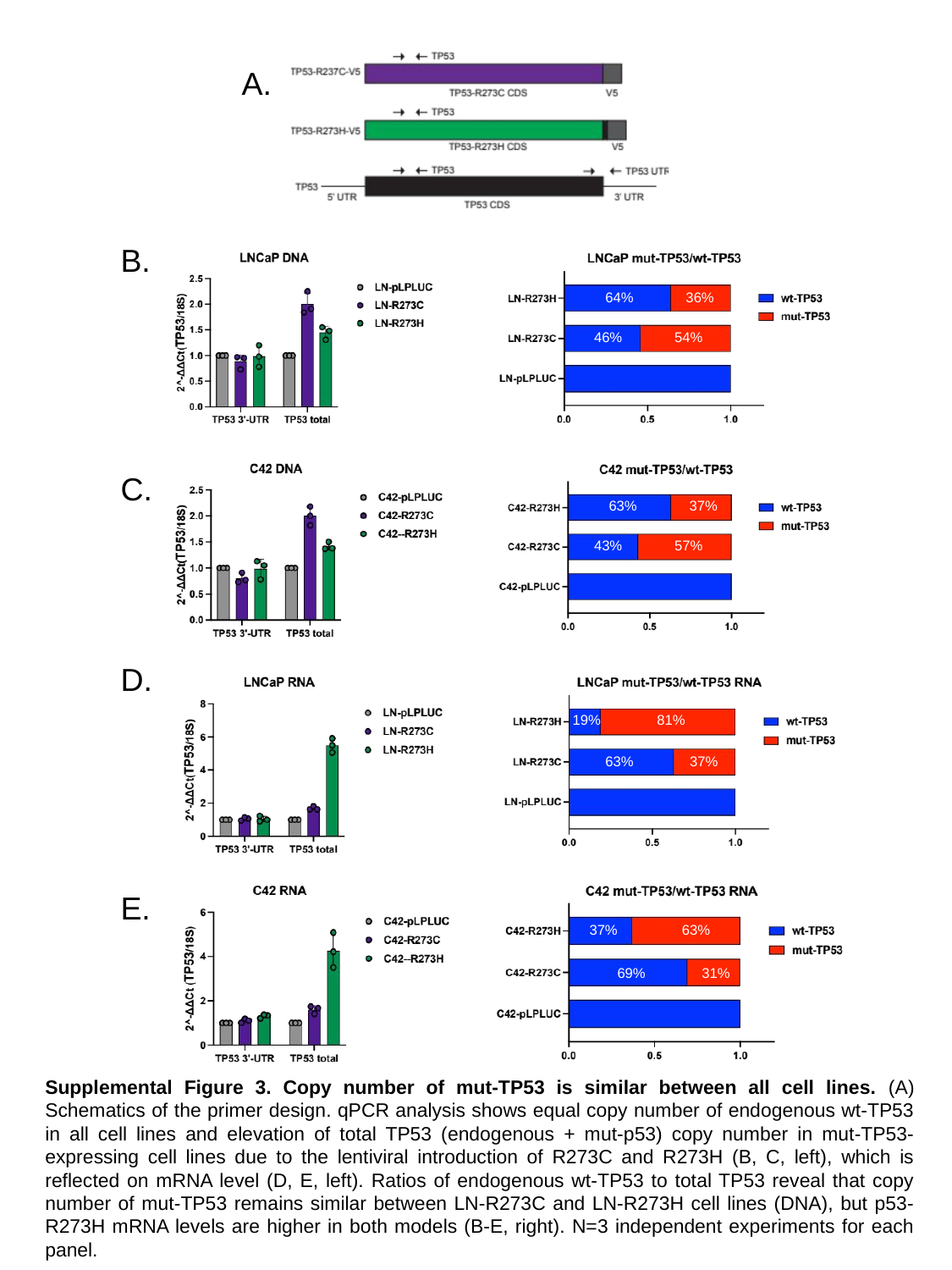

A.
B.
C.
D.
E.
 64% 36%
 46% 54%
 63% 37%
 43% 57%
19% 81%
 63% 37%
 37% 63%
 69% 31%
Supplemental Figure 3. Copy number of mut-TP53 is similar between all cell lines. (A) Schematics of the primer design. qPCR analysis shows equal copy number of endogenous wt-TP53 in all cell lines and elevation of total TP53 (endogenous + mut-p53) copy number in mut-TP53-expressing cell lines due to the lentiviral introduction of R273C and R273H (B, C, left), which is reflected on mRNA level (D, E, left). Ratios of endogenous wt-TP53 to total TP53 reveal that copy number of mut-TP53 remains similar between LN-R273C and LN-R273H cell lines (DNA), but p53-R273H mRNA levels are higher in both models (B-E, right). N=3 independent experiments for each panel.

## Slide 4
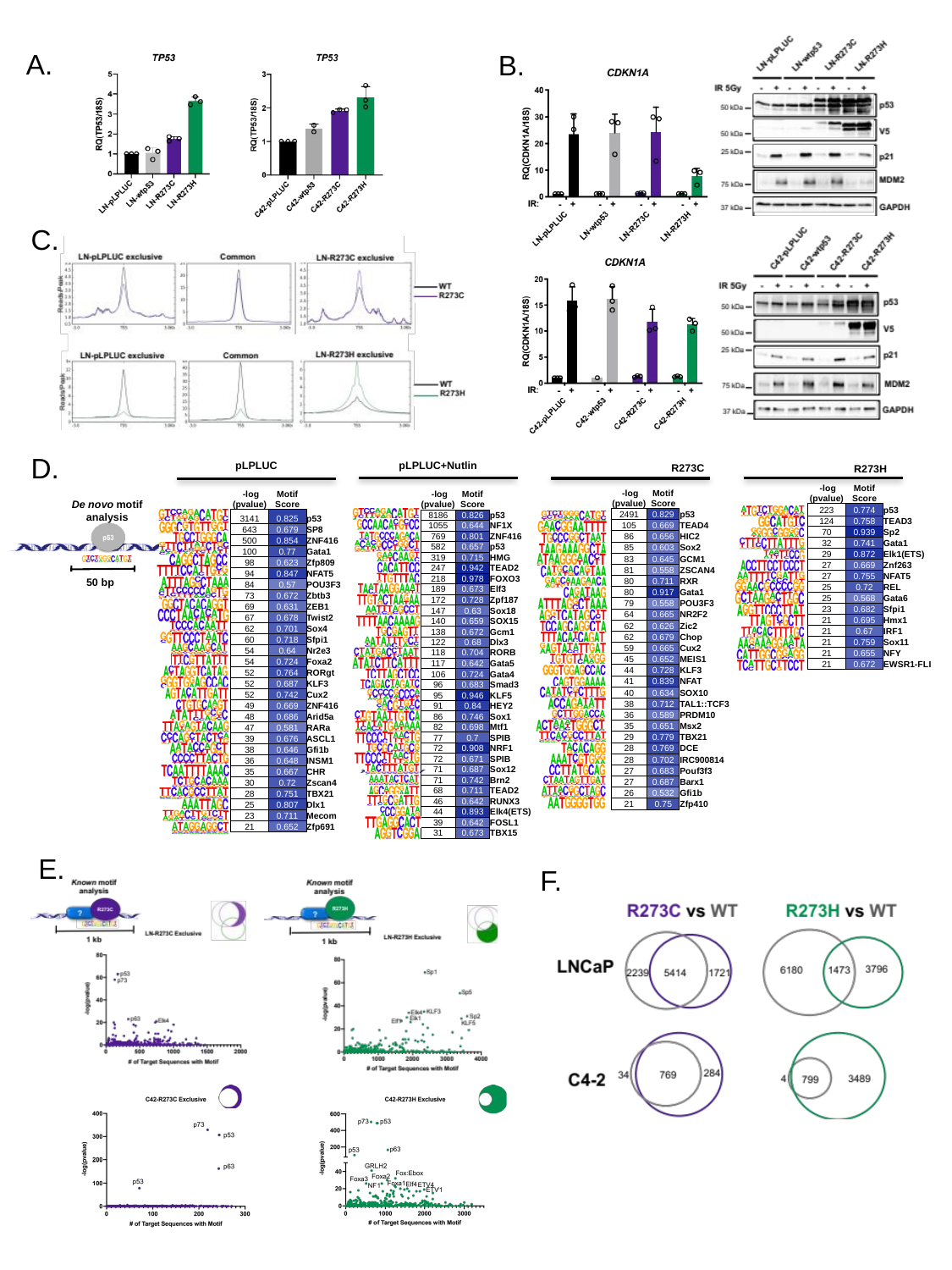

A.
B.
C.
D.
pLPLUC+Nutlin
pLPLUC
R273C
R273H
| -log (pvalue) | Motif Score | |
| --- | --- | --- |
| 223 | 0.774 | p53 |
| 124 | 0.758 | TEAD3 |
| 70 | 0.939 | Sp2 |
| 32 | 0.741 | Gata1 |
| 29 | 0.872 | Elk1(ETS) |
| 27 | 0.669 | Znf263 |
| 27 | 0.755 | NFAT5 |
| 25 | 0.72 | REL |
| 25 | 0.568 | Gata6 |
| 23 | 0.682 | Sfpi1 |
| 21 | 0.695 | Hmx1 |
| 21 | 0.67 | IRF1 |
| 21 | 0.759 | Sox11 |
| 21 | 0.655 | NFY |
| 21 | 0.672 | EWSR1-FLI |
| -log (pvalue) | Motif Score | |
| --- | --- | --- |
| 3141 | 0.825 | p53 |
| 643 | 0.679 | SP8 |
| 500 | 0.854 | ZNF416 |
| 100 | 0.77 | Gata1 |
| 98 | 0.623 | Zfp809 |
| 94 | 0.847 | NFAT5 |
| 84 | 0.57 | POU3F3 |
| 73 | 0.672 | Zbtb3 |
| 69 | 0.631 | ZEB1 |
| 67 | 0.678 | Twist2 |
| 62 | 0.701 | Sox4 |
| 60 | 0.718 | Sfpi1 |
| 54 | 0.64 | Nr2e3 |
| 54 | 0.724 | Foxa2 |
| 52 | 0.764 | RORgt |
| 52 | 0.687 | KLF3 |
| 52 | 0.742 | Cux2 |
| 49 | 0.669 | ZNF416 |
| 48 | 0.686 | Arid5a |
| 47 | 0.581 | RARa |
| 39 | 0.676 | ASCL1 |
| 38 | 0.646 | Gfi1b |
| 36 | 0.648 | INSM1 |
| 35 | 0.667 | CHR |
| 30 | 0.72 | Zscan4 |
| 28 | 0.751 | TBX21 |
| 25 | 0.807 | Dlx1 |
| 23 | 0.711 | Mecom |
| 21 | 0.652 | Zfp691 |
| -log (pvalue) | Motif Score | |
| --- | --- | --- |
| 2491 | 0.829 | p53 |
| 105 | 0.669 | TEAD4 |
| 86 | 0.656 | HIC2 |
| 85 | 0.603 | Sox2 |
| 83 | 0.645 | GCM1 |
| 81 | 0.558 | ZSCAN4 |
| 80 | 0.711 | RXR |
| 80 | 0.917 | Gata1 |
| 79 | 0.558 | POU3F3 |
| 64 | 0.665 | NR2F2 |
| 62 | 0.626 | Zic2 |
| 62 | 0.679 | Chop |
| 59 | 0.665 | Cux2 |
| 45 | 0.652 | MEIS1 |
| 44 | 0.728 | KLF3 |
| 41 | 0.839 | NFAT |
| 40 | 0.634 | SOX10 |
| 38 | 0.712 | TAL1::TCF3 |
| 36 | 0.589 | PRDM10 |
| 35 | 0.651 | Msx2 |
| 29 | 0.779 | TBX21 |
| 28 | 0.769 | DCE |
| 28 | 0.702 | IRC900814 |
| 27 | 0.683 | Pouf3f3 |
| 27 | 0.687 | Barx1 |
| 26 | 0.532 | Gfi1b |
| 21 | 0.75 | Zfp410 |
| -log (pvalue) | Motif Score | |
| --- | --- | --- |
| 8186 | 0.826 | p53 |
| 1055 | 0.644 | NF1X |
| 769 | 0.801 | ZNF416 |
| 582 | 0.657 | p53 |
| 319 | 0.715 | HMG |
| 247 | 0.942 | TEAD2 |
| 218 | 0.978 | FOXO3 |
| 189 | 0.673 | Elf3 |
| 172 | 0.728 | Zpf187 |
| 147 | 0.63 | Sox18 |
| 140 | 0.659 | SOX15 |
| 138 | 0.672 | Gcm1 |
| 122 | 0.68 | Dlx3 |
| 118 | 0.704 | RORB |
| 117 | 0.642 | Gata5 |
| 106 | 0.724 | Gata4 |
| 96 | 0.683 | Smad3 |
| 95 | 0.946 | KLF5 |
| 91 | 0.84 | HEY2 |
| 86 | 0.746 | Sox1 |
| 82 | 0.698 | Mtf1 |
| 77 | 0.7 | SPIB |
| 72 | 0.908 | NRF1 |
| 72 | 0.671 | SPIB |
| 71 | 0.687 | Sox12 |
| 71 | 0.742 | Brn2 |
| 68 | 0.711 | TEAD2 |
| 46 | 0.642 | RUNX3 |
| 44 | 0.893 | Elk4(ETS) |
| 39 | 0.642 | FOSL1 |
| 31 | 0.673 | TBX15 |
De novo motif
analysis
50 bp
Known motif analysis
?
1 kb
E.
F.

## Slide 5
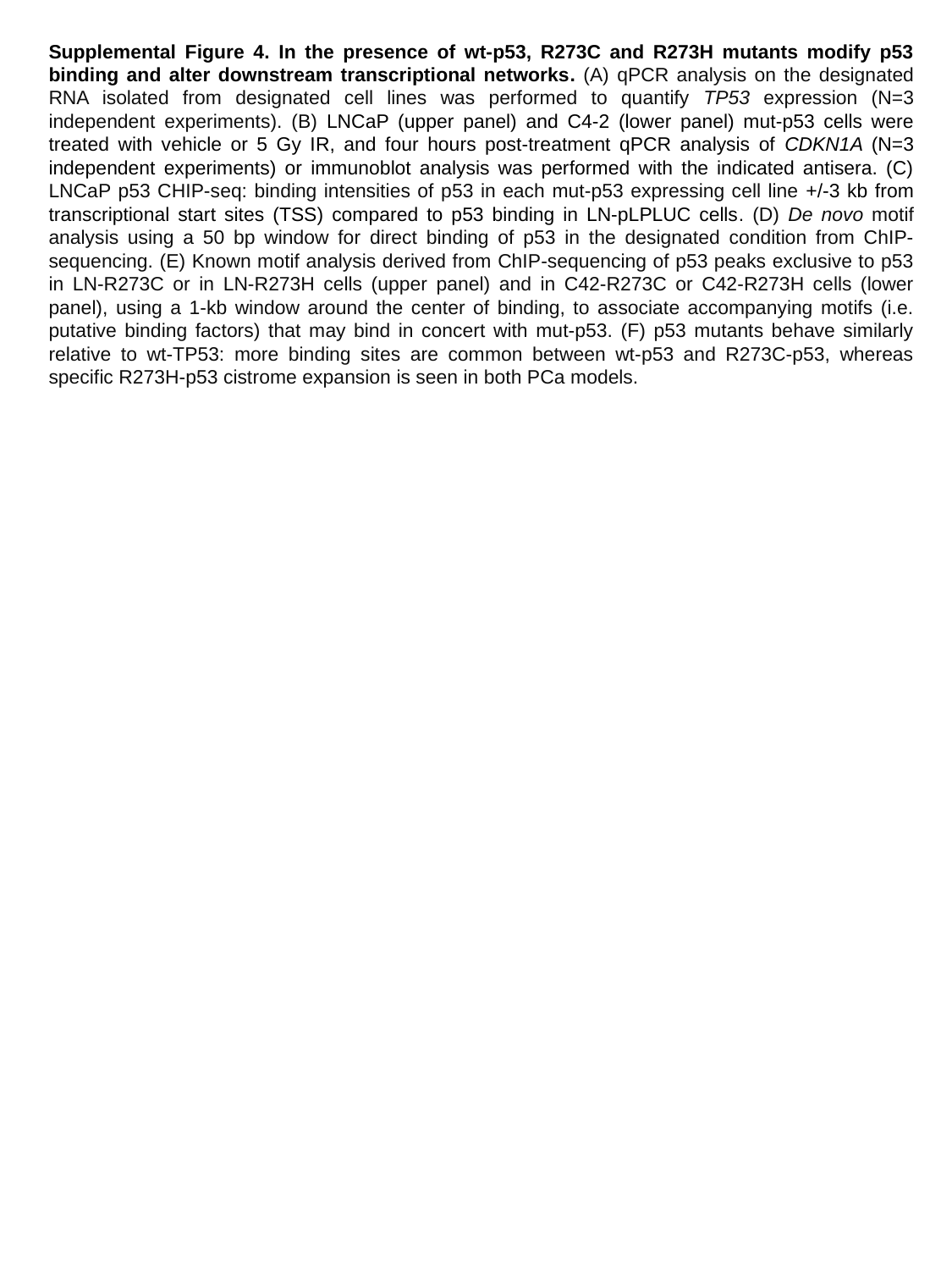

Supplemental Figure 4. In the presence of wt-p53, R273C and R273H mutants modify p53 binding and alter downstream transcriptional networks. (A) qPCR analysis on the designated RNA isolated from designated cell lines was performed to quantify TP53 expression (N=3 independent experiments). (B) LNCaP (upper panel) and C4-2 (lower panel) mut-p53 cells were treated with vehicle or 5 Gy IR, and four hours post-treatment qPCR analysis of CDKN1A (N=3 independent experiments) or immunoblot analysis was performed with the indicated antisera. (C) LNCaP p53 CHIP-seq: binding intensities of p53 in each mut-p53 expressing cell line +/-3 kb from transcriptional start sites (TSS) compared to p53 binding in LN-pLPLUC cells. (D) De novo motif analysis using a 50 bp window for direct binding of p53 in the designated condition from ChIP-sequencing. (E) Known motif analysis derived from ChIP-sequencing of p53 peaks exclusive to p53 in LN-R273C or in LN-R273H cells (upper panel) and in C42-R273C or C42-R273H cells (lower panel), using a 1-kb window around the center of binding, to associate accompanying motifs (i.e. putative binding factors) that may bind in concert with mut-p53. (F) p53 mutants behave similarly relative to wt-TP53: more binding sites are common between wt-p53 and R273C-p53, whereas specific R273H-p53 cistrome expansion is seen in both PCa models.

## Slide 6
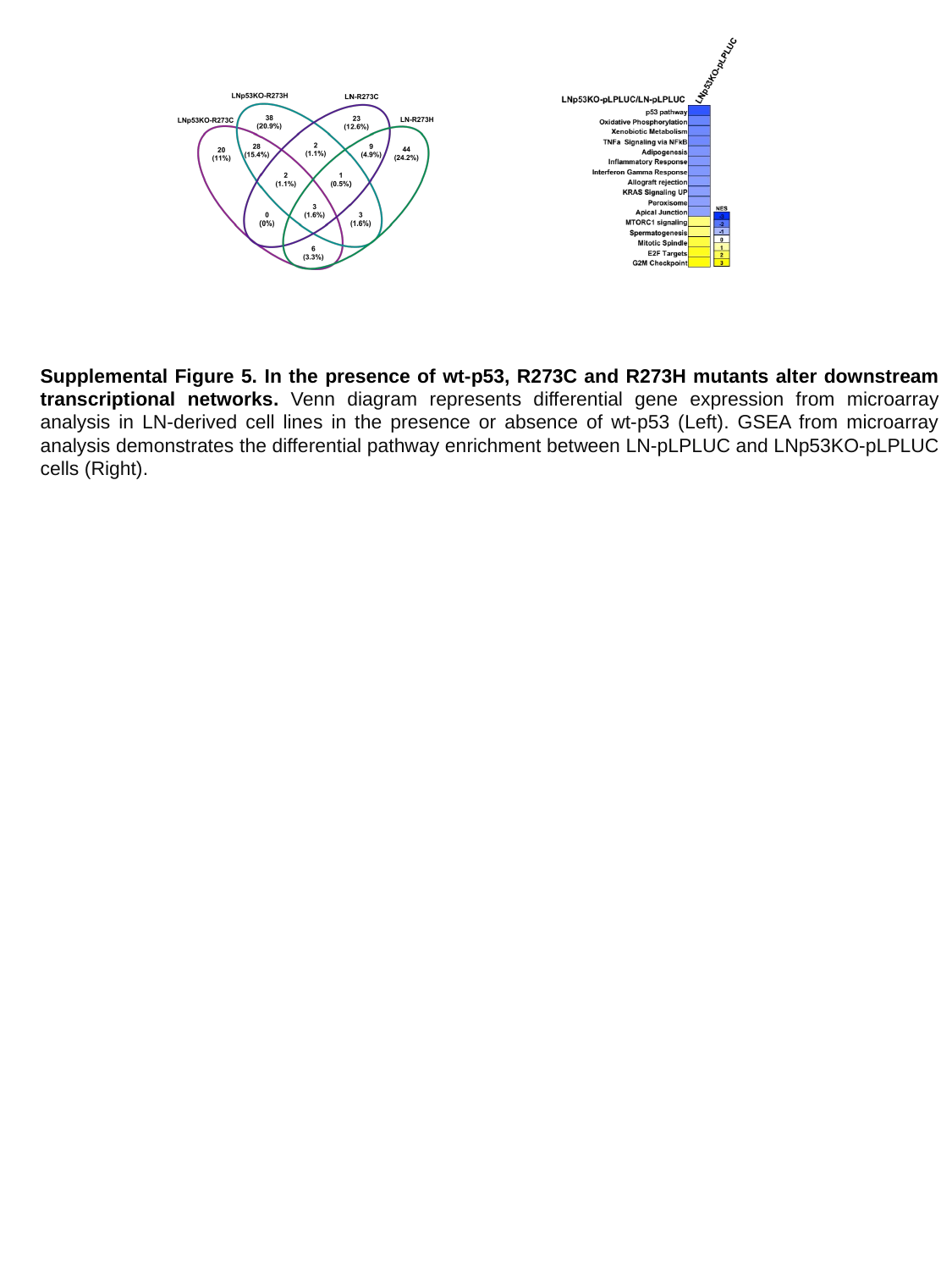

Supplemental Figure 5. In the presence of wt-p53, R273C and R273H mutants alter downstream transcriptional networks. Venn diagram represents differential gene expression from microarray analysis in LN-derived cell lines in the presence or absence of wt-p53 (Left). GSEA from microarray analysis demonstrates the differential pathway enrichment between LN-pLPLUC and LNp53KO-pLPLUC cells (Right).

## Slide 7
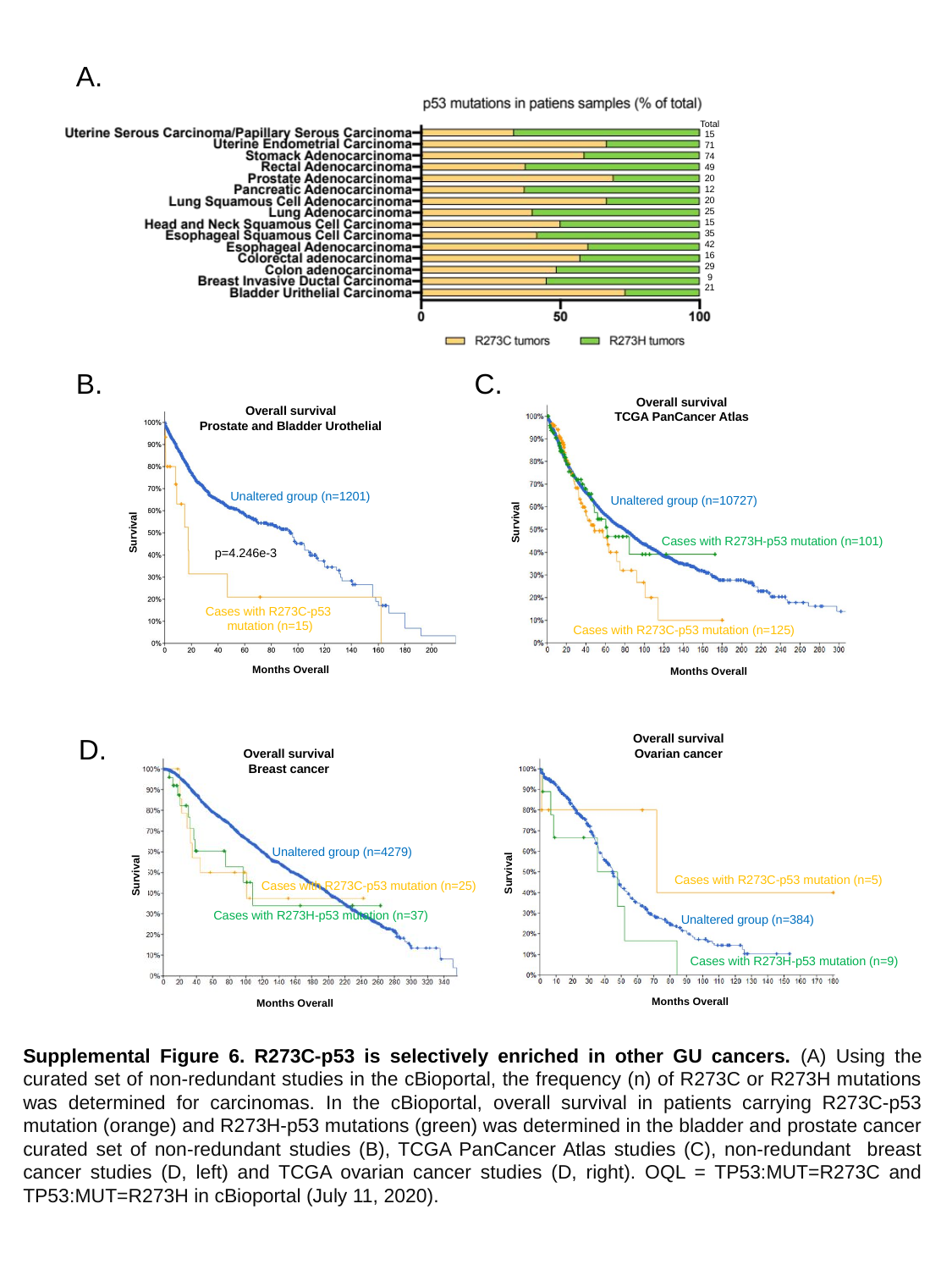

A.
Total
15
71
74
49
20
12
20
25
15
35
42
16
29
9
21
B.
C.
Overall survival
TCGA PanCancer Atlas
Unaltered group (n=10727)
Survival
Months Overall
Overall survival
Prostate and Bladder Urothelial
Unaltered group (n=1201)
Survival
Cases with R273H-p53 mutation (n=101)
p=4.246e-3
Cases with R273C-p53
mutation (n=15)
Cases with R273C-p53 mutation (n=125)
Months Overall
Overall survival
Ovarian cancer
Survival
Unaltered group (n=384)
Months Overall
D.
Overall survival
Breast cancer
Survival
Months Overall
Unaltered group (n=4279)
Cases with R273C-p53 mutation (n=25)
Cases with R273H-p53 mutation (n=37)
Cases with R273C-p53 mutation (n=5)
Cases with R273H-p53 mutation (n=9)
Supplemental Figure 6. R273C-p53 is selectively enriched in other GU cancers. (A) Using the curated set of non-redundant studies in the cBioportal, the frequency (n) of R273C or R273H mutations was determined for carcinomas. In the cBioportal, overall survival in patients carrying R273C-p53 mutation (orange) and R273H-p53 mutations (green) was determined in the bladder and prostate cancer curated set of non-redundant studies (B), TCGA PanCancer Atlas studies (C), non-redundant breast cancer studies (D, left) and TCGA ovarian cancer studies (D, right). OQL = TP53:MUT=R273C and TP53:MUT=R273H in cBioportal (July 11, 2020).
